# Comprehensive Profiling of the miRNome and Degradome Reveals Regulatory Signatures of Seed Aging and Germination

**DOI:** 10.3390/ijms26199292

**Published:** 2025-09-23

**Authors:** Marta Puchta-Jasińska, Paulina Bolc, Adrian Motor, Andreas Börner, Maja Boczkowska

**Affiliations:** 1 Plant Breeding and Acclimatization Institute-National Research Institute, Radzików, 05-870 Błonie, Poland; p.bolc@ihar.edu.pl (P.B.); a.motor@ihar.edu.pl (A.M.); 2 Leibniz Institute of Plant Genetics and Crop Plant Research (IPK), OT Gatersleben, Corrensstr 3, D-06466 Seeland, Germany; boerner@ipk-gatersleben.de

**Keywords:** germination, *Hordeum vulgare*, miRNA, degradome, viability

## Abstract

Small noncoding RNAs are recognized as crucial regulators of seed germination, but their role in seed aging remains unclear. To address this, we performed RNA sequencing (RNA-seq) on barley (*Hordeum vulgare* L.) seeds with varying viability levels after long-term storage in hermetically sealed containers since the 1972 harvest. This globally unique material, characterized by genetic homogeneity and contrasting germination capacities, enabled an in-depth analysis of microtranscriptomic changes during germination. We identified 62 known miRNAs from 11 families and 234 novel miRNAs, with miR159, miR168, and miR166 showing consistently high expression across all germination stages and viability groups. Differential expression analysis revealed 28 miRNAs whose abundance varied significantly with seed viability and germination phase. Functional predictions supported by quantitative reverse transcription PCR (qRT–PCR) and degradome-based target identification indicated that these miRNAs regulate key developmental and metabolic pathways. Several isomiRs exhibited sample-specific expression, suggesting the viability-dependent activation of distinct molecular mechanisms. Gene Ontology analysis highlighted processes related to nucleic acid binding, nuclear organization, and cytoplasmic metabolism as central during germination. We propose that miRNA profiles may reflect an “epigenetic inheritance”—a molecular memory of aging stored in seeds—rather than solely a response to current conditions. This concept may help explain aging-related phenotypes such as delayed germination and reduced vigor, warranting further investigation.

## 1. Introduction

Seed germination is a critical phase in the life cycle of seed plants, marking the transition from a dormant, environmentally resistant state to active growth and increased susceptibility. This process begins with water uptake (imbibition) and concludes with the emergence of the embryo axis, typically the radicle, preceding seedling establishment. Germination involves coordinated structural and metabolic changes that are essential for seedling development, including the activation of repair mechanisms that restore membranes, proteins, and DNA damaged during dehydration, storage, and rehydration [1,2]. Stored transcripts are translated early, followed by rapid activation of transcription, producing new mRNAs by the end of imbibition [3,4,5]. Cellular integrity is restored, respiration is initiated, and DNA repair, transcription, and protein biosynthesis are tightly linked to germination or dormancy status [6,7,8,9,10]. Nondormant seeds undergo additional processes, including cytoskeletal rebuilding, protein synthesis for embryo and root growth, and post-translational modifications that control protein stability [11].

Germination completion is defined by embryo protrusion through surrounding tissues (endosperm and testa), with the radicle typically emerging first. Despite extensive research, many molecular aspects remain unclear. Transcriptomic analyses have advanced the understanding of this phenomenon by revealing the coordinated regulation of transcription factors, hormonal signaling, energy metabolism, and stress responses during the transition from dormancy to active growth [12,13,14,15]. However, the molecular mechanisms underlying natural seed aging, especially during long-term storage when viability decreases, are less understood. Improving knowledge in this area is vital for managing gene bank resources.

Small noncoding RNAs, particularly microRNAs (miRNAs) and small interfering RNAs (siRNAs), are key endogenous regulators of gene expression in plants and act via sequence-specific gene silencing at the transcriptional and post-transcriptional levels. miRNAs, which are 19–24 nucleotides in length, can target multiple genes and regulate mRNA translation, cleavage, or poly(A) tail shortening [16,17,18]. These genes constitute approximately 1% of the predicted genes in higher eukaryotes and regulate 10–30% of gene expression. In plants, miRNAs control the development of roots, shoots, leaves, and flowers and play crucial roles in seed processes, including development, dormancy, and germination. Numerous miRNAs are implicated in stress responses, phytohormone signaling, antioxidant activity, and transcription factor regulation during germination. Nevertheless, the precise regulation of miRNAs and their targets during early germination remains incompletely understood and warrants further investigation.

Studies in *Arabidopsis thaliana* have demonstrated that abrupt changes in miRNA expression and interactions with trans-acting siRNAs (ta-siRNAs) regulate seed germination [19]. In radish, miR417 negatively affects germination under salinity stress, whereas miR395 acts as a positive regulator [20]. miR159 modulates gibberellin (GA) and abscisic acid (ABA) signaling, with its overexpression delaying germination [21]. The miR160 family targets auxin response factor (ARF) genes involved in auxin signaling, which are essential for plant growth and development [22]. The negative regulation of ARF10 by miR160 is critical for germination and postembryonic development via auxin–ABA pathway interactions. miR160 also modulates crosstalk among auxins, light, gibberellins, and brassinosteroids during hypocotyl elongation. miR417 likely regulates germination through ABA-dependent pathways, although its direct or indirect role remains unclear [23]. Additionally, several miRNAs (e.g., miR156, miR159, miR390, miR164, miR396, and miR319) regulate the ethylene and auxin signaling pathways during germination, influencing transcription factors such as ARF, MYB, SPL, NAC, and bHLH [16,21,24,25,26,27,28,29,30].

Seed aging is a natural, progressive, and irreversible process characterized by decreased viability and vigor, delayed germination, increased stress sensitivity, and eventual loss of germination capacity [31]. This deterioration results from physiological and biochemical changes, including reactive oxygen species (ROS) accumulation, lipid peroxidation, membrane damage, and protein and nucleic acid degradation. Seed senescence represents the terminal phase, involving irreversible cellular damage exceeding repair capacity, leading to programmed cell death. Maintaining seed viability is crucial for conserving plant genetic resources in gene banks, where orthodox seeds can remain viable for decades under optimal conditions, although longevity varies by species. Even under ideal storage, slow aging processes damage DNA, RNA, and cellular structures [32].

Recent studies have implicated miRNAs in seed viability and aging. In rice, upregulation of miR164c and downregulation of miR168a reduce viability and accelerate aging [33]. Differential expression of miR168 and miR817 was observed in artificially aged rice seeds, suggesting roles in seed vigor [34]. Conversely, natural aging in dry barley seeds did not alter the miRNA expression profile.

This study aimed to elucidate the role of miRNAs in the early germination stages of barley (*Hordeum vulgare* L.) seeds with varying viability due to long-term storage. Specifically, we sought to (I) identify miRNAs differentially expressed in embryos of high-[Hv], low-[Lv], and reconstituted-viability seeds [Rc] and (II) determine whether these miRNA expression patterns reflect the molecular consequences of seed aging. They are stored in dry, hermetically sealed containers. This unique material, which has a uniform genetic background and different germination capacities, provides pioneering information on changes in the microtranscriptome at various stages of germination. Furthermore, we investigated whether these miRNAs regulate genes involved in key pathways essential for germination, including signal transduction, phosphate metabolism, hormonal regulation, and the oxidative stress response. We hypothesized that long-term storage induces viability-dependent miRNA expression changes that serve as molecular signatures of aging. Differentially expressed miRNAs orchestrate transcriptional programs to restore metabolism and initiate germination, with degradome sequencing revealing critical miRNA targets in signaling and metabolic pathways governing germination capacity. Degradome analysis, also known as parallel analysis of RNA ends (PARE), is a high-throughput sequencing approach that enables the global identification of RNA cleavage sites, particularly those generated by miRNA-mediated transcript slicing. This method captures the 5′ ends of uncapped, degraded mRNA fragments, allowing the validation of predicted miRNA targets and providing direct evidence of post-transcriptional regulation.

## 2. Results

### 2.1. Overview of the Small RNA Library Sequence

Grains of *Hordeum vulgare* L. of the Damazy variety, which originated in Polanowice. Barley grains from the Plant Breeding Station, Rogaczewo, from the 1972 harvest were used for the study. For full postharvest maturity, the grains were stored three months after harvest at 18–25 °C. They were then dehydrated in laboratory vacuum dryers (SPT 200) under reduced air pressure (p = iTR) at 40 °C from a moisture content of approximately 15% to a final water content of 2.96%. To determine the effect of drying on grain viability, germination tests were conducted. Samples of grains with a viability above 95% were placed in airtight, air-filled flasks. The samples were stored for 42 years at ambient temperature. In 2015, germination capacity tests were conducted again. Seed samples with a high viability (Hv) of 83.3%, a moisture content of 3.58%, a low viability (Lv) of 2%, and a moisture content of 12.5% were selected for the tests that are the subject of this study. Seeds with high viability were propagated under field conditions in 2017/2018 and dried to 8% viability. The reproduced material (Rc) was used as a control sample in all the experiments described. Long-term storage seeds were imbibed for 24 h, and plant material for testing was collected at 6 h, 12 h and 24 h of imbibition. Research has focused on changes in the microtranscriptome of naturally aged seeds during germination. Degradome-seq analysis was performed to verify the target genes of the identified miRNAs. The NGS results were verified via qRT–PCR analysis (Supplementary File S8).

Two sequencing runs performed on the Illumina MiSeq platform using dry seed samples produced 28,019,900 and 25,796,300 raw reads, respectively. The samples were separated via the addition of six nucleotide barcodes to each library. The quality and length of the reads were filtered, with cutoff values of 17 bp and 25 bp, respectively. The number of reads obtained for renewed seeds (Rc, regenerated in the 2017–2018 season) averaged 815,627; for seeds with low viability (Lv, low viability; 2% germability after storage 1972–2018), 480,785; and for highly viable seeds (Hv, high viability; 86.7% germability after storage 1972–2018), 870,166. Over 40% of the reads were mapped to the *H. vulgare* reference genome, of which approximately 10% were rRNA and tRNA. Comprehensive details and descriptions of these sequencing runs for dry seeds have been reported by Puchta et al. [31]. Furthermore, three sequencing runs covering the nine stages during the germination process (Lv6, Lv12, Lv24, Hv6, Hv12, Hv24, Rc6, Rc12, Rc24) yielded 28,501,642, 29,253,255, and 27,424,301 raw reads, respectively (see Supplementary Files S2 and S9). The raw data were subjected to quality and length filtering, and sequences between 17 and 25 nucleotides were retained. After filtering, the proportion of high-quality reads averaged 45% for Rc samples, 39% for Hv samples, and 35% for Lv samples. Reads aligning to rRNA and tRNA sequences accounted for 0–3% of all the samples (Supplementary File S2). Genome-mapped reads constituted, on average, more than 70% of all samples. Further details about FastQC quality are presented in Supplementary File S9.

### 2.2. Identification of Known and Novel miRNAs in Barley During Germination

During germination, a total of 296 miRNAs were identified in the seed samples, including 62 known and 234 novel miRNAs. Among these, 20 known and 13 novel miRNAs were consistently present across all the samples and time points. Previously, 61 known and 81 novel miRNAs were reported in dry seeds [34].

In low-viability (Lv) seeds stored long term, 53 known and 144 novel miRNAs were detected during germination (Supplementary Files S3 and S4), with 37 known and 78 novel miRNAs common to all time points. In comparison, high-viability (Hv) seeds shared 30 known and 41 novel miRNAs, whereas regenerated (Rc) seeds had 41 known and 101 novel miRNAs in common.

Overall, 251 miRNAs (71 known, 180 novel) were expressed in high-viability seeds (Hv). Specifically, 21 known and 22 novel miRNAs appeared at 0 and 6 h, 26 known and 31 novel at 6, 12, and 24 h, and 21 known and 13 novel at 0 and 24 h.

**Figure 1:**
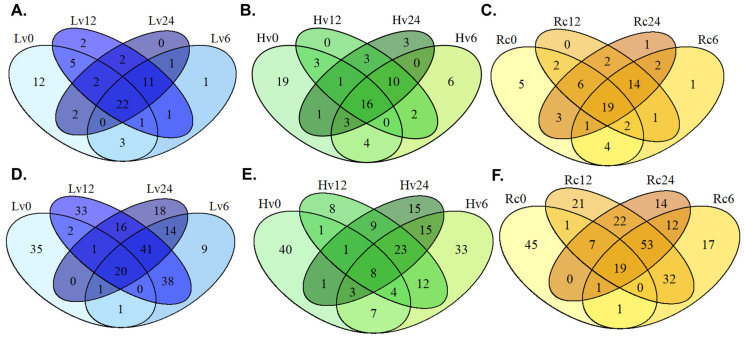
Venn diagram of several known and novel miRNAs detected in barley germination. (**A**) Known miRNAs detected in Lv—low-viability seeds after 0, 6, 12, and 24 h of germination; (**B**) known miRNAs detected in Hv—highly viable seeds after 0, 6, 12, and 24 h of germination; (**C**) known miRNAs detected in Rc—regenerated seeds after 0, 6, 12, and 24 h of germination; (**D**) new miRNAs detected in Lv—low-viability seeds after 0, 6, 12, and 24 h of germination; (**E**) new miRNAs detected in Hv—highly viable seeds after 0, 6, 12, and 24 h of germination; (**F**) new miRNAs detected in Rc—regenerated seeds after 0, 6, 12, and 24 h of germination.

The regenerated seeds (Rc) presented the greatest miRNA diversity, with 33 known and 19 novel miRNAs detected at all three germination stages (6, 12, and 24 h). Additionally, 26 known and 21 novel miRNAs were unique at 0 and 6 h, and 29 known and 27 novel miRNAs were unique at 0 and 24 h. The detailed data are shown in Figure 1.

In the analyzed miRNA libraries, miRNAs ranged in length from 18 to 25 nucleotides (bp). During germination (6, 12 and 24 h), the most prevalent known miRNAs measured 21 nb, followed closely by 20 bp miRNAs. Known miRNAs of 22 bp were the least common, and no known miRNAs longer than 22 bp were observed in the samples.

**Figure 2:**
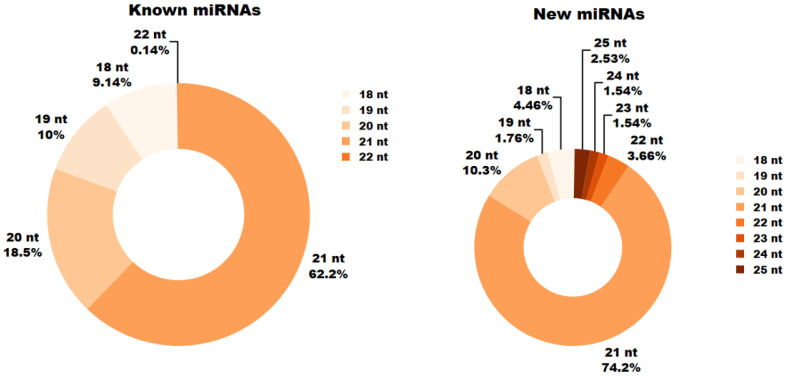
The percentage size distribution of known and novel microRNAs has been identified via next-generation sequencing. The experiment involved RNA sequencing of barley seeds at 6, 12, and 24 h intervals after germination. The colour variation across the scale denotes the length of the identified microRNA sequences.

For novel miRNAs, the greatest numbers were found at lengths of 21 bp and 20 bp, whereas only 2.53% of new miRNAs measured 25 bp (Figure 2).

### 2.3. Expression Profiles of miRNAs at Different Germination Stages

**Figure 3:**
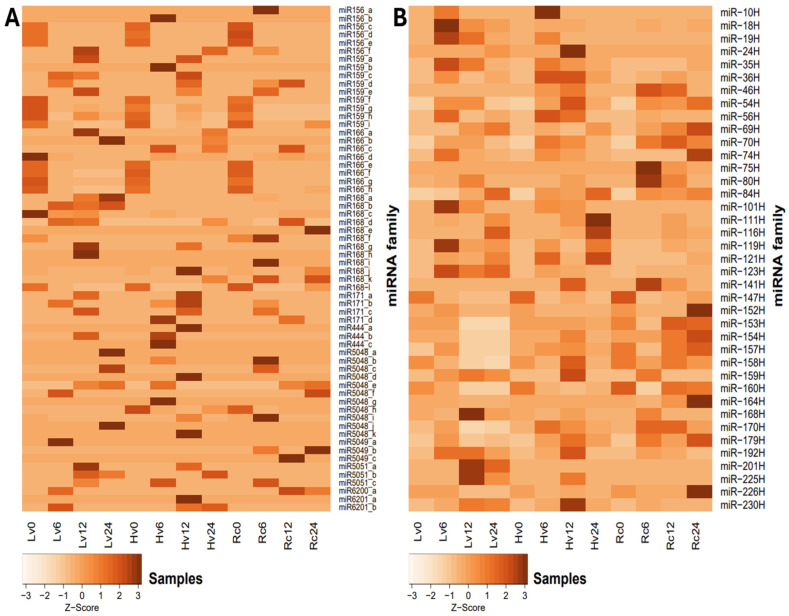
The heatmap shows the miRNAs with the highest level of significance in terms of differential expression at 0, 6, 12, and 24 h of germination. (**A**) known miRNA; (**B**) novel miRNA. The Z-score indicates row-wise standardization: for each miRNA, expression values across time points are centered to the mean and scaled by the standard deviation. The colors reflect relative expression dynamics: dark orange indicates greater-than-average expression (positive Z-scores), and white indicates lower-than-average expression (negative Z-scores) relative to the mean miRNA score.

Families with constant expression levels were observed regardless of germination time or sample viability. However, individual isomiRs presented greater variation in expression levels between samples (Figure 3A). Among the miRNA families analyzed, miR159, miR168, and miR166 presented the highest expression levels overall. Notably, the expression of miR168 peaked at 8,243.8 revolutions per minute (RPM) in the Rc6 sample (Supplementary File S3). The expression of the miR6200 family was lower, with 1.3 RPM in Rc12 and 21.59 RPM in Hv0 (Supplementary File S3). Conversely, miR5049 presented the highest expression at the initial time point (0 h) across all the samples. After 24 h of germination, miR5049 expression was measured at 3.47 RPM in Rc and 2.0 RPM in Hv6. miR-1120 was exclusively detected at 0 h in all samples, whereas miR397 appeared only in Hv0. In addition to miR168, the greatest expression of most miRNA families was observed in dry seeds (0 h). Further details are provided in Figure 3. The highest level of miR156_a expression was observed in Rc6, reaching 7.1 RPM, with only low expression detected elsewhere, except in Rc24 (1.4 RPM). miR159_a was strongly expressed specifically in Lv12 (180.5 RPM), whereas miR159_h was highly expressed in Hv12 (3040.0 RPM). miR166_c exceeded 2000 RPM in Rc12, and miR168_b exhibited elevated expression only in Hv24 (93.3 RPM). Additionally, miR444_a and miR5048_k were uniquely expressed in Lv12 at 1.4 RPM and 0.7 RPM, respectively, whereas miR5049_b was detected solely in Lv24 (1.4 RPM). Only 11 novel isomiRs were unique to dry seeds before imbibition, whereas 16 isomiRs from 12 known miRNA families (including miR156, miR159, miR166, miR168, miR444, miR5048, miR5049, and miR6200) were not expressed in dry seeds (0 h).

Focusing on novel isomiRs with expression above 1 RPM (Figure 3B), miR-10H appeared only in Hv6 (1.19 RPM) and Lv6 (2.82 RPM). miR-19H was exclusive to Hv samples, with expression ranging from 1.20 to 8.91 RPM across germination stages. miR-154H was highly expressed (>50 RPM) in most samples except Hv12 and Hv24 (~3 RPM).

miR-160H was detected in the Hv and Lv samples at 6 h (14.67 and 11.70 RPM, respectively), at 12 h in Rc12 (35.27 RPM) and Lv12 (6.08 RPM), and at 24 h in Rc24 (31.42 RPM) and Lv24 (10.29 RPM). miR-201H was present only in Hv12 (4.11 RPM) and Hv24 (2.54 RPM) (Supplementary Files S3 and S4).

Differential expression (DE) analysis was conducted to compare miRNA levels in barley seeds with varying viability over 24 h of germination. Ten miRNAs (six novel, four known) were selected for RT–qPCR validation, and their expression patterns were consistent with the NGS data. For example, miR-191H and miR-225H were detected in Hv12 and Hv24, whereas miR-56H was detected in Hv6 and Hv24. miR159g, miR166e, miR166f, and miR156c were expressed across all samples and time points. miR-10H was specific to Lv and Hv at 6 h. (Figure 3).

**Figure 4:**
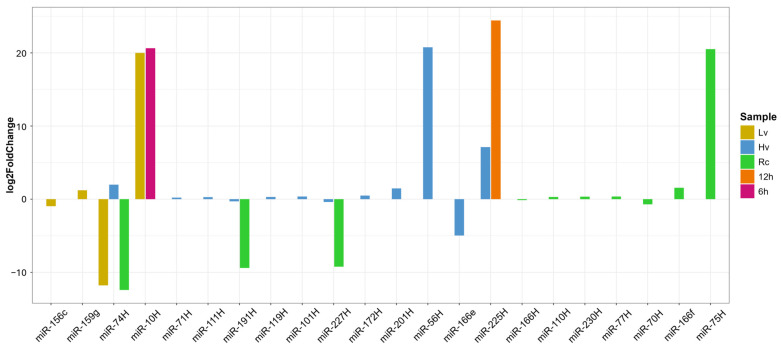
Identification of differentially expressed downregulated and upregulated miRNAs in seed samples: low viability (Lv); high viability (Hv); regenerated seeds (Rc); and after 6 h of imbibition (6 h) and 12 h of imbibition (12 h). Differential expression analysis was performed with DESeq2 via a negative binomial generalized linear model.

DEG analysis was performed via two approaches: (1) comparing viability groups (Hv, Lv, Rc) at each time point and (2) assessing expression changes within samples over time (0, 6, 12, 24 h). In Rc seeds, ten miRNAs showed significant changes—five upregulated (e.g., miR-75H, miR-10H, and miR-56H) and five downregulated (e.g., miR-74, miR-191H, and miR-227H) (Figure 4). In Hv seeds, miR-56H had the greatest increase, whereas miR166_e was the most downregulated. Lv seeds presented three miRNAs whose expression significantly changed over time; notably, the expression of miR-10H and miR-225H was strongly upregulated at 6 and 12 h (Figure 4).

### 2.4. Target Prediction and Validation for miRNA-Mapped Cleavage Sites

Degradome sequencing revealed 288 sequences corresponding to 151 distinct miRNAs.

In the low-viability (Lv) group, 43 degradome sequences were identified: 2 at 6 h, 16 at 12 h, and 25 at 24 h. The high-viability (Hv) samples contained 107 sequences distributed as follows: 27 at 6 h, 47 at 12 h, and 33 at 24 h. The regenerated (Rc) samples presented 138 sequences, with 31 at 6 h, 56 at 12 h, and 51 at 24 h.

**Figure 5:**
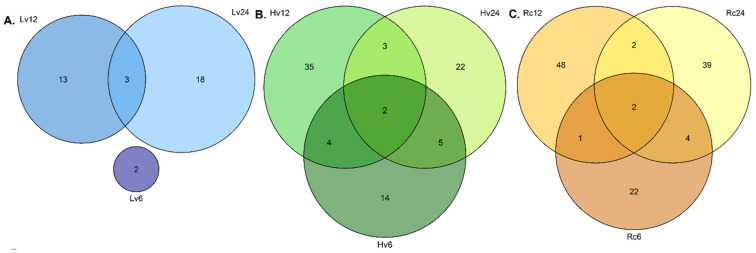
(**A**) Venn diagram of degradome sequences detected in Lv—low-viability seeds after 6, 12, and 24 h of germination. (**B**) Degradome sequences detected in Hv—high-viability seeds after 6, 12, and 24 h of germination. (**C**) Degradome sequences in Rc—regenerated seeds after 6, 12, and 24 h of germination, as determined via CleaveLand4 software.

The number of unique degradome sequences increased over time in the Lv samples, with 2, 13, and 18 unique sequences detected at 6, 12, and 24 h, respectively (Figure 5). Three sequences were shared between Lv12 and Lv24. In the Hv samples, unique sequences from 14 at 6 h to 35 at 12 h were observed. The Rc samples presented 22 unique sequences at 6 h, increasing to 48 at 12 h and 39 at 24 h (Figure 5).

**Figure 6:**
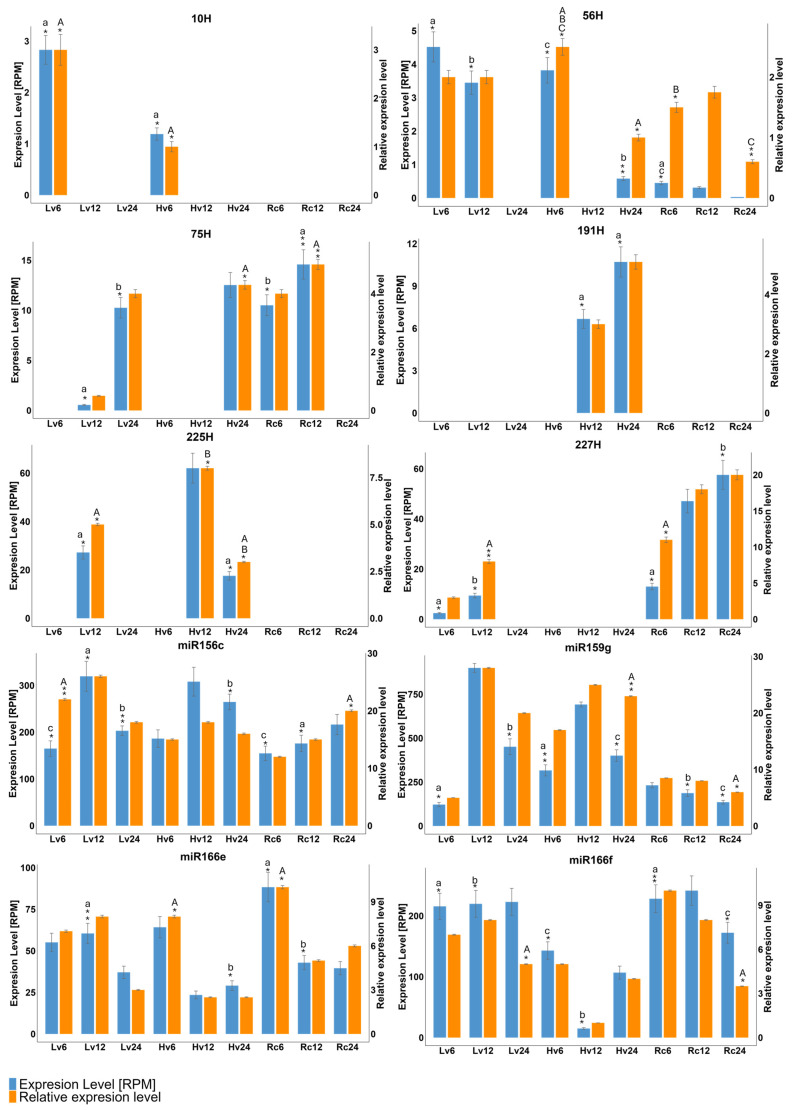
The expression levels of a selected differentially expressed microRNA (DEG) in barley were validated at 6, 12, and 24 h following seed germination using both quantitative reverse transcription PCR (qRT-PCR) and small RNA sequencing (sRNA-Seq) methodologies. QRT-PCR subsequently quantified the relative expression values. Each analysis was conducted with three biological replicates and three technical replicates per sample. The data are expressed as the mean ± standard error of the mean (SEM). The differential expression analysis was conducted using DESeq2 with a false discovery rate (FDR) threshold of <0.05. The selected microRNAs identified as differentially expressed were then subjected to further validation through quantitative real-time polymerase chain reaction (qRT-PCR). Statistical significance is indicated by asterisks, where ** *p* < 0.01, and * *p* < 0.05, determined by one-way analysis of variance (ANOVA) Letters (a–c) above the bars indicate results of anova testing for qRT-PCR and (A–C) for RNA-Seq data.

Quantitative reverse transcription PCR (qRT–PCR) confirmed the highest expression of miR156c and miR159g in the Hv12 samples, followed by that of miR166f in the Rc6 and Rc12 samples. Novel miR-10H was detected only in Lv6 and Hv6, whereas miR-191H and miR-225H were detected in Hv12 and Hv24. The expression of miR-75H peaked in the Lv12, Lv24, Hv24, Rc6 and Rc12 samples (Figure 6).

**Figure 7:**
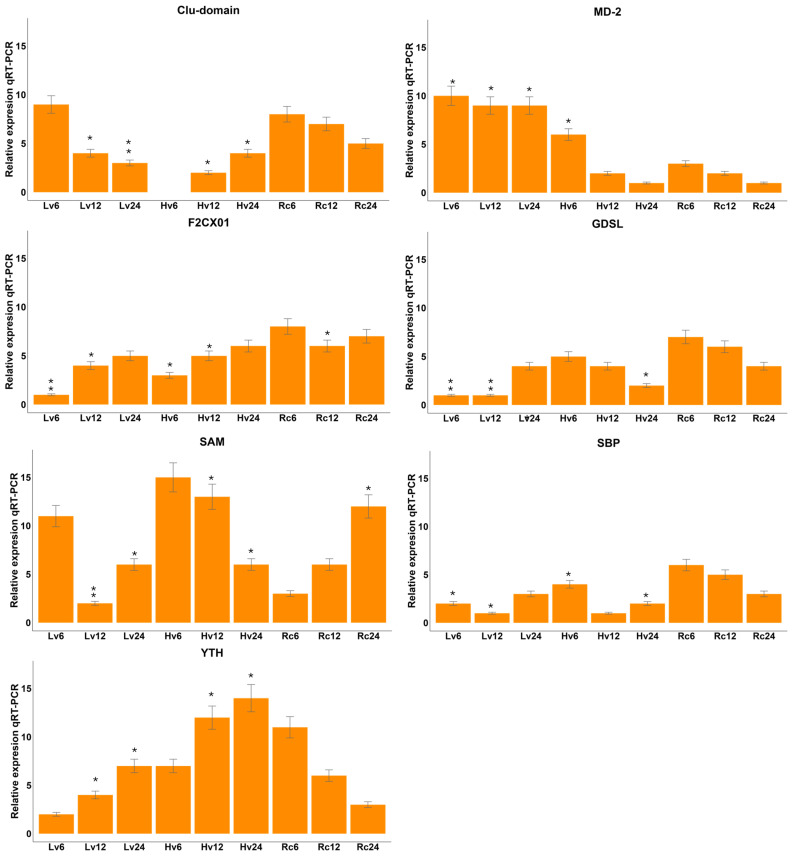
The expression levels of selected target genes regulated by differentially expressed microRNAs (DEG miRNAs) in barley were validated at 6, 12, and 24 h post-germination using both quantitative reverse transcription PCR (qRT-PCR) and small RNA sequencing (sRNA-Seq) approaches. QRT-PCR subsequently quantified the relative expression values. Each experiment included three biological replicates, with three technical replicates per sample. The data are expressed as the mean ± standard error of the mean (SEM). The differential expression analysis was conducted using the DESeq2 method with a false discovery rate (FDR) cutoff of <0.05. A select group of microRNAs was validated by implementing quantitative real-time polymerase chain reaction (qRT-PCR). Asterisks indicate statistical significance: The results of the one-way analysis of variance (ANOVA) revealed a probability value of ** *p* < 0.01, and * *p* < 0.05, respectively. The regenerated sample (Rc) was used as the comparative expression analysis control.

Targets related to significant miRNAs identified via degradome-seq analysis were verified via qRT–PCR analysis. Degradome sequencing was performed to validate the predicted miRNA–target interactions experimentally. This approach captures the 5′ ends of cleaved mRNAs, enabling the identification of miRNA-guided slicing events in vivo. Using CleaveLand4, we mapped degradome reads to the barley transcriptome and identified specific mRNA targets cleaved in a miRNA-dependent manner. These results confirmed the functional activity of several differentially expressed miRNAs by providing direct evidence of their target cleavage. The F2CXO1 gene presented the highest level in the Rc samples and the lowest expression in the Lv6 samples (Figure 7). The F2CXO1 gene presented the highest expression level in the Rc samples and the lowest in the Lv6 samples (see Figure 7). The MD-2 gene presented the highest expression level in the Lv6 and Lv12 samples; the lowest was detected in the Hv12 sample. The GDSL gene presented the highest expression in the Rc and Hv samples, whereas it was expressed at low levels in the Lv samples. The highest expression of the SAM gene was observed in samples Hv6, Hv12, and Rc24, whereas the lowest expression was observed in samples Lv12 and Rc6. The SBP gene presented very low expression in all the samples. The highest expression of the YTH gene was observed in samples Hv12, Hv24, and Rc6. The observed expression levels were inversely proportional to the expression of the corresponding miRNA. The observed expression levels were inversely proportional to the expression of the corresponding microRNA.

### 2.5. Functional Annotation of the Predicted Targets

To explore the potential functions of the identified sequences, Gene Ontology (GO) enrichment analysis was conducted. The Rc6 sample presented the greatest number of associations with biological processes and molecular functions that were absent in the other germination stages and samples (Figure 7). No significant GO terms (*p* < 0.01) were found in the Rc12, Rc24, or Lv6 samples.

In the Lv12 sample, sequences were linked to various activities and processes, including phosphomannomutase activity; 3-isopropylmalate dehydrogenase activity; biosynthesis and metabolism of alpha-amino acids, branched-chain amino acids, and carboxylic acids; and small molecule biosynthesis, pyruvate family amino acid metabolism, proline metabolism, and organic acid biosynthesis.

For Hv6, enriched functions involved oxidoreductase activity targeting donor CH-OH groups with NAD/NADP as acceptors, NAD binding, magnesium ion binding, small molecule biosynthesis, pyruvate family amino acid metabolism, biosynthesis of proteinogenic amino acids, and L-leucine metabolism.

In Hv24, the sequences were associated with 3-isopropylmalate dehydrogenase activity, branched-chain amino acid biosynthesis, L-leucine metabolism, pyruvate family amino acid metabolism, and small molecule biosynthesis.

**Figure 8:**
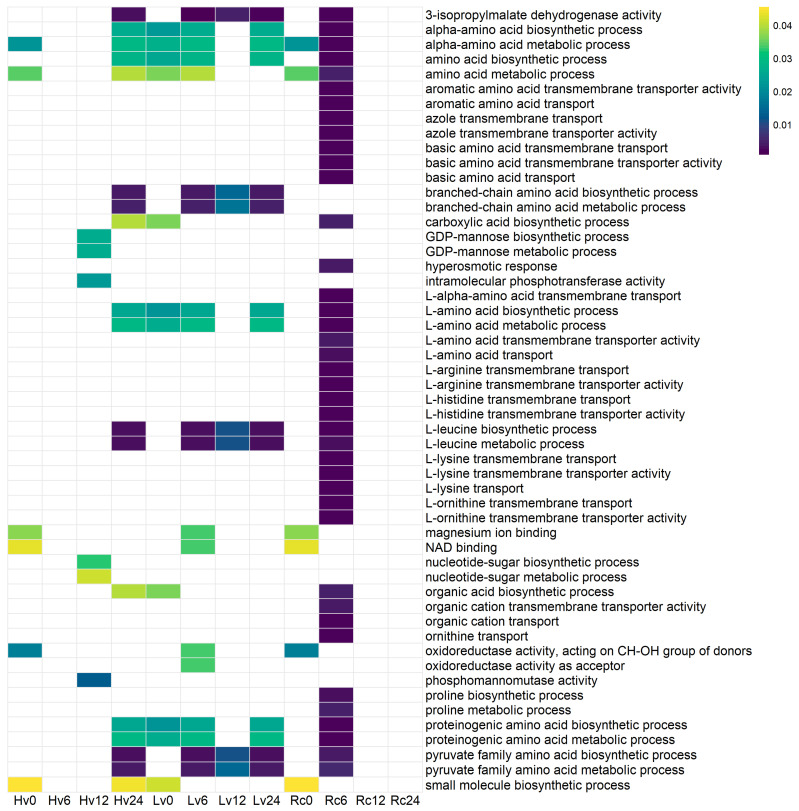
GO functional analysis of DEGs for degradome sequences correlated with miRNAs, with significantly different expression identified by DeSeq2 analysis of Lv (low viability), Hv (high viability), and Rc (regenerated) seed samples. iRNAs were identified as differentially expressed via DESeq2 (FDR < 0.05), and enriched GO biological processes (*p* < 0.01, FDR-corrected) were identified as confirmed targets. Functional enrichment analysis of these miRNA-correlated DEGs was performed via the g:Profiler web server.

The functions of target genes and their corresponding proteins were examined for miRNAs exhibiting statistically significant expression differences between samples with identifiable degradome sequences (Figure 8).

**Figure 9:**
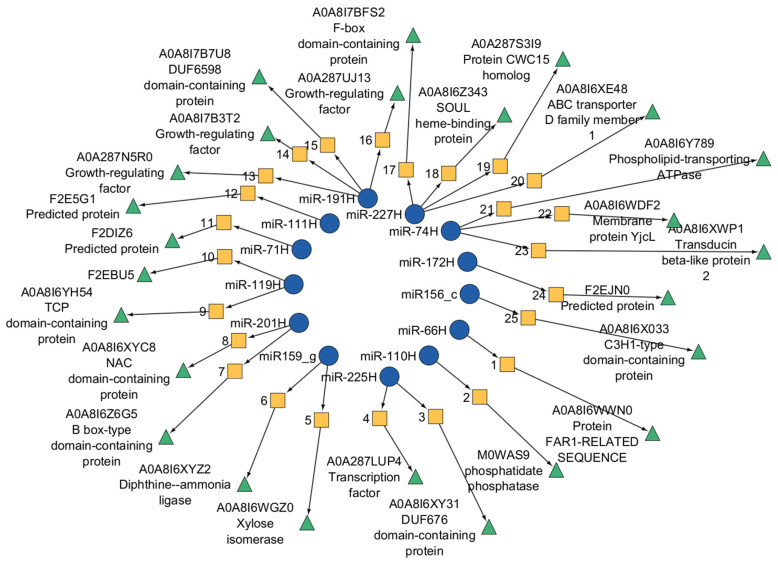
Protein annotation (green triangle) of the target gene sequence (yellow square) of miRNAs (blue circle) that are differentially expressed in the samples. The protein numbers refer to the UniProt database. For more details about the target gene sequence, see Supplementary Files S6 and S7.

In low-viability (Lv) seeds at 12 h after germination, three target genes encoding a DUF676 domain-containing protein, an NAC domain-containing protein, and a chloramphenicol acetyltransferase-like domain 1 protein were detected (Figure 9). By 24 h, additional targets had emerged, including genes encoding xylose isomerase, a zinc finger C2H1 domain-containing protein, a FAR-1-related protein, DUF676 domain-containing protein, phospholipid-transporting ATPase, NAC domain-containing protein, TCP domain-containing protein, and B box-type domain-containing protein. No targets were identified at 6 h in the Lv samples.

In highly viable samples, target genes related to diphthine ammonia ligases and F-Box domain-containing proteins were identified at 6 h. At 12 h, the miRNAs regulated genes encoding MYB, DUF676 domain-containing protein, and F-box domain-containing protein. Additional targets included proteins with transmembrane helices, mitochondrial carrier domain 2, and transducin beta-like protein 2.

For the Hv samples at 6 h, significant expression changes corresponded to phosphatidate phosphatase family proteins, F-box domain-containing proteins, and ABC transporter D family member 1. At 12 h, the SOUL heme-binding protein and aquaporin-like protein TIP-11 were targeted. After 24 h, the targets included the membrane protein YjcL, DUF6598 domain-containing protein, growth-regulating factor, and protein CWC15 homolog.

## 3. Discussion

### 3.1. Role of miRNAs in Germination

Small noncoding RNAs are crucial regulators of seed germination and plant development. To date, changes in the miRNome during the germination of long-term stored seeds have not been explored. This study utilized unique plant material derived from a single long-term stored seed batch, ensuring that the observed differences were not influenced by environmental variability.

During extended storage, one sample was unintentionally unsealed, resulting in increased seed moisture and a significant decline in viability. This naturally occurring variation in seed vigor enabled the investigation of how viability levels affect the germination process by comparing seeds with differing capacities to germinate.

This research enhances our understanding of how prolonged storage and related physiological changes impact the activity of small RNAs during germination. By using seeds from the same batch that differed only in vigor, the relationship between seed viability and changes in miRNome expression was precisely determined.

### 3.2. Stability of miRNA Versus Susceptibility of Other RNA Fractions to Degradation in Dry Seeds

The decline in seed viability is associated with changes at the macromolecular level. Reactive oxygen species are believed to be responsible for these alterations and may also disrupt the germination process [35]. Damage affects proteins as well as DNA and RNA. Among these macromolecules, RNA is the most susceptible to degradation during seed aging; as viability decreases, both the total RNA content and RNA integrity decrease [34,36,37,38,39]. The stability of miRNAs results from their incorporation into the RISC, which physically protects them [40].

According to data from the EMBL-EBI Expression Atlas, RISCs are highly expressed during the development and germination of barley seeds [40]. On this basis, it can be assumed that the initial miRNA content was similar across all long-term stored seed samples analyzed in this study. The differences observed during germination most likely reflect seed aging processes and variations in germination capacity.

### 3.3. Families of miRNAs and Their Specific Roles During Germination

Small noncoding RNAs play essential roles in germination and plant development [41]. Our findings revealed distinct miRNA expression patterns in seed samples with varying viability during the first 24 h of germination. The observed differences in expression highlight the dynamic nature of miRNA-mediated regulation in response to seed viability and germination timing (Supplementary File S5).

In this study, we identified 62 known miRNAs from 11 different families, along with 234 novel miRNAs. Regardless of seed viability or germination stage, 20 miRNAs were consistently present across all the samples. The known families included miR156, miR159, miR166, miR168, miR171, miR5048, and miR5051. As reported by Puchta et al. [34], these families were also detected in the dry seeds of the studied samples.

The miR156, miR159, miR166, miR168, and miR171 families are highly conserved among land plants and are present in both monocots and dicots [42]. These genes are generally highly expressed, suggesting important roles in metabolic processes essential for normal development and function.

miR156 is highly conserved in plants and targets the plant-specific transcription factor *Squamosa promoter binding protein-like* (*SPL*). It regulates age-dependent development and responses to biotic and abiotic stimuli [43,44].

Only the miR156a isoform was detected in dry barley seeds with varying viabilities. It presented comparable expression levels across all the samples and had the highest abundance among all the identified miRNAs [34]. During germination, miR156a was present only in fresh seeds after 6 h, with negligible expression in the other samples (Rc6; 7.1; Supplementary File S3). The high abundance of miR156a transcripts in dry seeds and their presence during early germination of the Rc sample suggest a role in initiating and properly regulating the germination process.

In seeds with low viability, miR156b predominated, and other isoforms—with the exception of miR156a—were present during early germination stages. The expression of miR156f was high at later stages in fresh seeds. It regulates plant architecture by targeting *OsSPL7*, influencing shoot number and height, and downregulating the tillering regulators *Teosinte Branched 1* (*TB1*) and *Lax Panicle 1* (*LAX1*) [45,46].

High miR156f expression during early germination likely supports normal coleoptile growth and prevents premature tillering. This may contribute to the increased number of abnormal seedlings observed in low-viability seeds [47,48,49].

miR159 is an evolutionarily conserved microRNA family found in most land plants. It targets GAMYB transcription factors, which are essential for grain development, germination, and anther formation [50,51,52,53,54,55]. GAMYB inhibits growth in vegetative tissues, so miR159 silences GAMYB, promoting vigorous growth [35].

During the early development of barley seeds, miR159a is expressed at low levels and is present in all dry seeds regardless of their viability [34,56]. However, during germination, up to nine isoforms of miR159 were detected, with this family showing one of the highest expression levels in the early stages. The expression of these genes remained relatively stable across all samples and viability levels (Figure 5), although previous studies indicated a gradual increase during germination and growth [23,57].

High expression of miR159 may regulate the activation of starch reserves through gibberellin signaling mediated by the GAMYB factor, which induces α-amylase and hydrolase enzymes in aleurone cells, as well as programmed cell death [58,59]. Unlike barley, mature miR159 is absent in rice seeds but is upregulated in *Arabidopsis* seeds in response to stress and hormones such as ABA and drought [21,26]. Similarly, miR159 accumulates under drought conditions in maize, wheat, and barley, suggesting a role in enhancing stress tolerance. This may explain its high early expression during barley seed germination [18].

The expression of miR168, a highly conserved microRNA family in higher plants, is high during the early germination stage of barley seeds, whereas its transcript levels are significantly lower in dry seeds [34,60]. It regulates AGO1, a key protein involved in post-transcriptional gene silencing, thereby modulating the activity of all miRNAs through the RISC. *AGO1* homeostasis is maintained by miR168, which directs the cleavage of AGO1 transcripts and stabilizes *AGO1* through its interaction [61,62,63].

Germination activates genes involved in cellular repair, metabolism, and reserve utilization, which are regulated at multiple levels, including by miRNAs [64]. The expression of miR168 and its isoforms is significantly greater in imbibed seeds than in dry seeds [34]. Both mature miR168a-5p and miR168a-3p were detected in dry seeds, which is consistent with findings in rice, maize, and barley [34,57]. miR168a was detected only in high-viability seeds after 12 h and in regenerated seeds after 6 h. It was absent in low-viability seeds and in the early germination stages of high-viability seeds (Supplementary File S3).

Twelve miR168 isoforms were identified, making it the most diverse miRNA family detected. Compared with dicots, monocots show greater diversity and more AGO1 members [65]. miR168 expression was the lowest in low-viability seeds, suggesting elevated AGO1 levels and intensified post-transcriptional regulation during early germination in these seeds. Further RNA-seq analysis is needed to confirm this hypothesis.

Deng et al. (2016) [66] attributed the high expression of miRNAs to their role in barley growth and development. They observed variability in the number of identified miRNAs, ranging from just a few to thousands. Their conclusions align with our observations.

Differences in miRNA family expression levels likely reflect the activity of various physiological and biochemical processes occurring in seeds during imbibition [67]. Variation in individual miRNA expression patterns may indicate which pathways are activated during germination. Consistent expression trends were observed via both qRT–PCR and next-generation sequencing (NGS) analyses.

In this study, we identified 234 novel microRNAs. Research on barley seed development and germination has revealed numerous new miRNAs. A common characteristic of these novel microRNAs is their low expression levels [23,68]. This finding aligns with other studies, although discrepancies may arise from differences in methodological sensitivity [69].

### 3.4. Functional Analysis of miRNA-Regulated Genes in Seeds with Varying Viability

Germination requires dry seeds to resume metabolism and initiate changes at the transcriptional level. Analysis of proteins correlated with high seed viability revealed their involvement in membrane transport, DNA-related transcriptional changes, and cell differentiation.

Proteins of the MYB family play key roles in regulating germination. Garcia-Gimez et al. [66] demonstrated that MYB factors influence β-glucan accumulation in cell walls, which is correlated with membrane permeability during germination. In the early stages of *Arabidopsis* seed germination, MYB proteins regulate this process and interact with abscisic acid (ABA) signaling pathways [21,70]. Similar regulatory roles were observed during sheepgrass germination under drought stress [71].

Proteins with an F-box domain participate in proteasomal ubiquitination. For example, the expression of the OsFbx352 protein in germinating rice is upregulated by ABA and inhibited by glucose, suggesting that F-box proteins modulate glucose-regulated inhibition of seed germination by altering ABA metabolism [72].

Proteins with the DUF676 domain, which is part of the serine esterase family, contribute to signaling defense responses. Ortiz et al. [69] highlighted their key role in green algae, whereas Gao et al. [70] classified DUF domain proteins identified in the mosses *Oryza sativa*, *Zea mays*, *Hordeum vulgare*, and *Aquilegia coerulea* as coparticipants in pathogen response pathways [73,74].

Proteins associated with miRNA target genes that show significant expression differences in high-viability seeds may indicate the initiation of metabolic processes. These processes likely involve alterations in cell wall permeability and the hormonal regulation necessary for breaking seed dormancy.

In seeds with low viability, the DEG miRNAs were associated with proteins responsible for xylose isomerase activity, the C3H1 domain (involved in DNA and RNA binding), the TCP domain I, the B-box domain, and the phospholipid-transporting ATPase, as reported in a study on *Davidia involucrata Baill*.

The involvement of proteins with the C3H1 domain in long-term seed dormancy was observed. Similarly, delayed root development and silique deformation have been observed in *Arabidopsis* and tobacco. This study demonstrated that DIZF proteins with a C3H1 domain negatively regulate root growth and development and prolong anthesis [75].

CaKR1, a C3H1 domain protein identified in *Capsicum annuum*, functions as a multifunctional stress-responsive protein involved in plant defense and environmental adaptation pathways [76]. C3H1-type zinc finger domains, including those in *PEI1*, which plays a role in embryogenesis, have been identified in several *Arabidopsis* genes. Although the exact function of the zinc finger motif remains unclear, some members regulate RNA stability by binding pre-mRNAs and modulating transcription [77].

Acid phosphatase group proteins have been extracted from seeds of lentil [78], pumpkin [79], soybean [79], and castor bean [80]. The enzymatic activity profiles of fresh, stored, and aged seeds are similar, suggesting that the enzyme structure is preserved during storage and plays a critical role in maintaining seed viability [81].

NAC proteins are induced under aerobic conditions in roots, shoots, and germinating seeds and are associated with reduced germination capacity. These genes have been identified in *Arabidopsis* and *Petunia* under low-oxygen conditions and are known to adversely affect drought stress tolerance. NAC proteins likely participate in the low-oxygen response through interactions with ABA and ABI [82].

In *Arabidopsis*, NAC103 responds to ABA during germination, resulting in non-ABA sensitization and a faster onset of germination. These results suggest that NAC positively regulates ABA [28]. Some NAC proteins are membrane-bound, and their controlled proteolytic activation is proposed as an adaptive strategy that enables rapid transcriptional responses to sudden environmental changes [83].

The correlations found in low-viability seeds between proteins involved in membrane transport, root growth, and NAC proteins suggest that metabolism resumes in these seeds, although the process may not be completed successfully. Previous studies on dry-stored barley seeds have demonstrated the initiation of metabolic processes regulated by miRNAs, including organic cation transport, amino acid biosynthesis, transmembrane transport, oxidoreductase activity, and NAD binding. This could be related to increased seed moisture [34].

Damage to transcripts stored in low-viability seeds could underlie difficulties in completing germination properly [39].

### 3.5. Key miRNA-Regulated Processes in Phosphorylation and Phosphorus Metabolism During Germination

**Figure 10:**
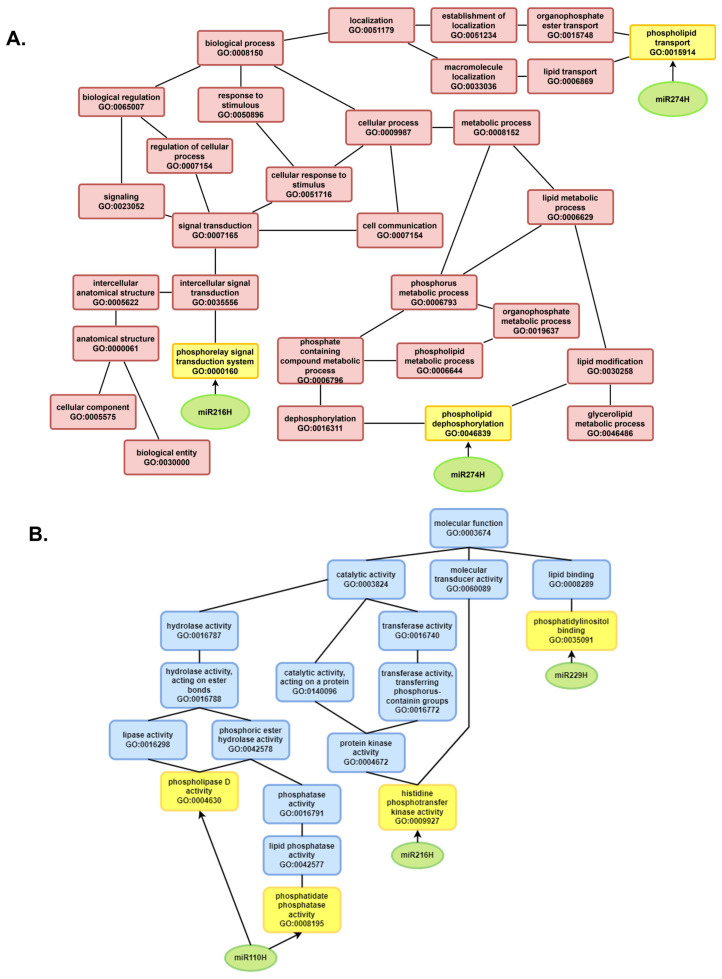
(**A**,**B**) Phosphorylation and phosphorus metabolism pathways identified in the GO annotation of miRNAs related to degradome sequences. The GO annotation diagram was created via the G:Profiler algorithm. The black lines between the GO terms represent relationships between terms. In G:profiler, the child term represents a more specific subclass of the parent term, corresponding to an ontological relationship such as ‘is a’ or ‘part of’, as commonly defined in biological ontologies. [A child term in Gene Ontology represents a more specific biological concept that falls under the broader category of its parent term].

The increased expression and diversity of miRNAs during germination compared with dry seeds indicate their crucial role in regulating processes essential for resuming metabolism. Functional analysis of genes regulated by known miRNAs revealed that, regardless of the seed viability level, these genes primarily control protein modifications, especially phosphorylation, a reversible post-translational modification (Figure 10).

Kinases add a phosphate group to a protein, a fundamental process in signal transduction and enzyme regulation. Phosphorylation activates or deactivates proteins within signaling pathways, enabling plants to respond to environmental factors and hormonal signals during germination (Figure 10). It also alters enzyme activity, thereby controlling metabolic pathways necessary for energy production and biosynthesis [84].

Although the mechanisms remain incompletely understood, studies suggest that the association of different miRNAs with AGO proteins during RISC loading depends on phosphorylation. The integration of miRNA biogenesis into cellular responses to stress and development occurs through the transcriptional regulation of individual *MIR* loci by phosphoproteins, such as hyponastic leaf 1 (HYL1) in the DCL1 complex [85]. Consequently, miRNAs that regulate phosphorylation can influence the biogenesis, activity, and efficiency of other miRNAs during seed germination.

Across all the seed samples, miRNA-regulated genes were also involved in the metabolism of phosphorus- and phosphate-containing compounds. Phosphorus plays a critical role in energy transfer and signal transduction. It is also essential for synthesizing nucleic acids and membranes, making it vital for plant growth and development.

During seed germination, phosphorus and phosphate metabolism are crucial for numerous biochemical and physiological changes required to transition from dormancy to active growth. These include energy transfer, nucleic acid synthesis, phospholipid biosynthesis, and signal transduction.

Adenosine triphosphate (ATP) is produced through cellular respiration during germination and serves as an energy source for various metabolic activities. The hydrolysis of ATP to adenosine diphosphate (ADP) releases energy that drives the biochemical reactions necessary for seedling growth [86,87,88].

Phosphorus is an essential component of the backbone of DNA and RNA molecules. Its availability is vital for the synthesis of new nucleic acids during germination, which support cell division and protein synthesis [89]. Nucleotides, which depend on phosphorus, are fundamental for genetic material, act as energy carriers (e.g., ATP, GTP), and serve as cofactors in enzymatic reactions [90].

Moreover, phosphorus is a key element in the head groups of phospholipids and plays a critical role in maintaining membrane integrity and functionality. The synthesis of new membranes is essential for cell expansion and the formation of new cellular structures during germination.

Protein deterioration is a primary factor affecting seed longevity. Proteins perform multiple functions in seeds, including structural support and enzyme activity. Damage to proteins can result from oxidation, deamination, glycation, and fragmentation. Such damage impairs seed germination by inactivating enzymes and damaging cellular structures, leading to reduced seed vitality [91,92,93].

These findings suggest that miRNAs may play a key role in regulating mechanisms that repair protein damage caused by seed aging.

### 3.6. Association of microRNA and Degradome Profiles with the Physiological Status of Barley Seeds

This study employed an omics approach to identify molecular correlations associated with three classes of barley seed viability differentiated by germination retention after long-term storage. Analysis of the miRNAome and degradome revealed specific patterns of miRNA expression and transcript degradation that may suggest differential activation of metabolic pathways, stress response mechanisms, and developmental processes during germination.

The most incredible diversity of miRNAs and the most pronounced degradation signals within categories 0–2 were observed in the case of Rc seeds regenerated in the field from the basal sample. This finding indicates increased activity of the RNA-induced silencing complex (RISC) and active silencing of target transcripts. The substantial number of identified degradome sequences in Rc12 and Rc24 (Figure 10B), along with the presence of miRNAs that regulate phosphorylation and signal transduction, correlates with the physiological viability of these seeds and their ability to fully reactivate metabolic processes. These findings align with the Rc seed phenotype, which is characterized by high germination energy and synchronized embryo growth.

In Hv seeds stored under high viability conditions, the miRNA profile suggests partial activation of metabolic pathways. This was accompanied by a reduced number of unique degradome sequences and decreased expression of key miR168 and miRNA156 isoforms. Notably, the presence of miRNAs involved in regulating oxidative stress responses, as well as those modulating ARF and MYB transcription factors, suggests the activation of compensatory mechanisms in response to storage-induced damage. The expression of these regulatory miRNAs may explain why Hv seeds retain partial germination capacity despite molecular signs of deterioration.

In contrast, Lv seeds presented severely reduced viability. These genes were characterized by a markedly diminished number of active miRNAs, fewer unique degradome sequences, and miRNAs indicative of activation in pathways related to residual metabolism or apoptosis. Degradomes assigned to category 0 were detected in the Lv12 and Lv24 samples; however, their proportion was substantially lower than that in the Rc seeds, suggesting insufficient activation of the transcript silencing machinery. Concurrently, the presence of miRNAs that target NAC proteins, TCP transcription factors, and membrane transporters may reflect ineffective attempts to reactivate metabolic processes amid extensive cellular damage.

The differences in the miRNA profiles and degradomes among the seed groups suggest that the miRNAome may serve as a molecular indicator of seed viability, reflecting the integrity of regulatory systems and metabolic potential. MiRNAs such as miR168, miR159, and miR166, along with their isoforms, may function as molecular biomarkers of seed functional status in response to long-term storage. The expression of these genes is correlated with, and likely contributes to, the observed differences in germination rates and efficiencies among the Rc, Hv, and Lv groups.

### 3.7. Differences in miRNA Expression and Target Genes Between High- and Low-Viability Barley Seeds

Comparative analysis of Hv (high viability) and Lv (low viability) seeds revealed distinct patterns of miRNA expression and target degradation that reflect fundamentally different physiological states during germination. In Hv seeds, which retained partial viability despite prolonged storage, the miRNA expression profile suggested partial activation of key metabolic pathways. This was supported by the presence of miRNAs involved in redox balance and hormone signaling (e.g., ARF- and MYB-regulating miRNAs), which may serve as compensatory mechanisms in response to storage-induced oxidative stress [70,94]. Additionally, the downregulation of miR168 and miR156 isoforms—known regulators of AGO1 and SPL transcription factors, respectively—may indicate an altered regulatory landscape that allows seeds to bypass or delay typical germination checkpoints [16].

Although the number of unique degradome sequences in Hv seeds was reduced, functional GO enrichment analyses still identified targets involved in energy metabolism, amino acid biosynthesis, and molecular transport. This implies that, despite signs of molecular deterioration, Hv seeds retain enough regulatory capacity to initiate germination, albeit less efficiently [95].

In stark contrast, Lv seeds presented a markedly impaired molecular signature. A lower number of active miRNAs, fewer unique degradome sequences, and the near absence of enriched GO terms at early time points (Lv6) suggest widespread regulatory failure. The few miRNAs detected were predominantly linked to residual metabolic activity or stress-related pathways, such as apoptosis or senescence. Interestingly, degradome category 0 targets—representing high-confidence miRNA cleavage events—were observed only at later time points (Lv12 and Lv24) and at lower proportions than Rc (regenerated) seeds did, indicating that the transcript-silencing machinery may have been activated too late or insufficiently to rescue cellular function [96,97].

Moreover, the presence of miRNAs that target transcription factors such as NAC and TCP domain proteins, as well as membrane transporters, in Lv samples suggests ineffective or abortive attempts to restore regulatory control and reinitiate metabolism under conditions of extensive cellular damage [28]. These findings are consistent with prior research showing that loss of small RNA regulation is associated with seed deterioration, compromised stress responses, and reduced germination capacity [91,98].

Taken together, these results highlight a critical role for miRNA–mRNA interactions not only in regulating germination-associated processes but also in buffering the molecular consequences of seed aging. The differential degradome and expression profiles between Hv and Lv seeds may serve as early biomarkers of seed vigor and could inform strategies to improve seed longevity through biotechnological or storage-based interventions.

## 4. Materials and Methods

### 4.1. Plant Material

**Figure 11:**
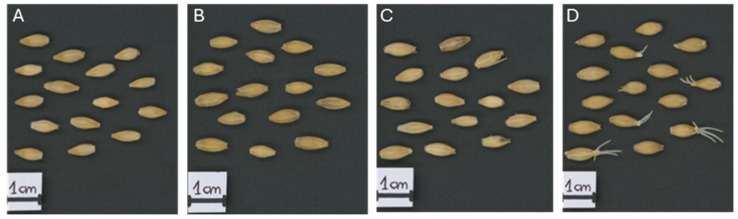
*Hordeum vulgare* grains at (**A**) 0 h, (**B**) 6 h, (**C**) 12 h and (**D**) 24 h of imbibition.

Barley (*Hordeum vulgare* L., cv. Damazy) seeds harvested in 1972 were stored in airtight containers at room temperature. Seed viability and moisture content were assessed in 2015, and low-viability (Lv; 2% viability, 12.5% moisture) and high-viability (Hv; 84% viability, 4.36% moisture) groups were distinguished. Seeds from the same original batch, maintained under gene bank conditions (99.3% viability, 6.32% moisture in 2015), were field propagated in 2019 and used as a reference. [Detailed sample information is available in Puchta et al. 2021 for each group [34], and embryos with scutella were collected at 6, 12, and 24 h after imbibition in triplicate (25 embryos per replicate)]. The Lv seeds had a germination index (GI) of 0.53 and a germination rate (SE) of 0.1% after 24 h, whereas the Hv values were 33.3 and 47%, respectively. The Rc seeds presented the highest GI (42.4) and SE (59%) values (Figure 11 and Supplement File S8).

### 4.2. miRNA Extraction and Construction of sRNA Libraries

Embryonic tissues (25 seeds per sample) were separated into three biological replicates and ground in liquid nitrogen. From each of the tested seed groups with different viabilities (Rc, Hv, Lv) after 6, 12, and 24 h, 24 embryos were isolated in 3 replicates. Isolation and library construction were performed for each replicate. Replicates were not pooled at any stage of the analysis. miRNA was isolated via a MicroRNA kit (A&A Biotechnology, Gdynia, Poland). miRNA was quantitatively evaluated via a Nanodrop 1000 spectrophotometer (Thermo Fisher Scientific, Waltham, MA, USA) and agarose gel electrophoresis. To confirm the results and evaluate miRNA integrity, a 2100 Bioanalyzer with a Small RNA Kit (Agilent Santa Clara, Santa Clara, CA, USA) was used. Small RNA libraries were prepared from three biological replicates of isolates via the NebNext Multiplex Small RNA Library Prep Set for Illumina (New England BioLabs, Ipswich, MA, USA). The miRNA libraries were cleaned of postreaction elements with Monarch Kits for RNA Cleanup Kit (New England BioLabs, Ipswich, MA, USA). Size selection was performed with a Pippin prep 3% agarose gel cassette (Sage Science, Beverly, MA, USA). The final step in the process involved the sequencing of the microRNA on the MiSeq instrument (Illumina) using Reagent Kits v2 (50-cycles) in the 51 single-end mode.

The extraction of the microRNA fractions and the preparation of the sRNA libraries were carried out in accordance with the methods described in the relevant literature. A total of 27 libraries were subjected to sequencing on the Illumina MiSeq platform (51 bp, single-end reads, 150 cycles). The minimum coverage of 2 million sRNA reads was utilised for each sample. sRNA-Seq libraries were prepared in triplicate for each of the research samples

### 4.3. Degradome Sequencing

Total RNA was extracted using TRIzol (Invitrogen, Waltham, MA, USA). The mRNA fraction was isolated from the total RNA with magnetic beads, and 5′ adapters were ligated to the 3′ mRNA cleavage products. cDNA was synthesized using customer-provided primers and reagents from the NEBNext Small RNA Library Prep Kit (New England Biolabs, Ipswich, MA, USA). The subsequent phase entails the digestion of samples with the restriction enzyme Mme I, which is employed to generate uniform 3′ ends. This is followed by the ligation of a double-stranded 3′ adapter and the PCR amplification using primers compatible with Illumina sequencing platforms.

The next step involves the purification of libraries and the size selection of these libraries using the Pippin Prep 3% Agarose Gel Cassette (Sage Science, Beverly, MA, USA). The final quality of the libraries was evaluated using the Agilent Bioanalyzer 2100 High Sensitivity DNA Analysis and the Qubit dsDNA HS Assay Kit (Thermo Fisher Scientific, Waltham, MA, USA). The libraries were then sequenced using the Illumina NovaSeq (Illumina, San Diego, CA, USA) with 150 base pairs of paired-end reads. The comprehensive protocol for the preparation of the degradome-seq library, adapted for seeds exhibiting low viability, is delineated in the publication by Puchta et al. (2025) [99].

### 4.4. Bioinformatic Analysis

The quality of the reads obtained from the sRNA-Seq and degradome-Seq sequencing was assessed via FastQC software (version 0.12.1) [47]. The removal of adapters from the raw reads was performed via the UEA Small RNA Workbench: Adapter Removal software. Low-quality reads (Q < 30) and reads shorter than 17 bp and longer than 25 bp were subsequently filtered via UEA Small RNA Workbench Filter software (version 4.4) [48]. The high-quality sequences were then mapped to the *Hordeum vulgare* reference genome (MorexV3_pseudomolecules_assembly), which was obtained from the Ensembl Plants database (Release 57, accessed on 17 February 2023) [49]. The known miRNAs and their isoforms were identified through a search of the miRBase database version 22.1 [50]. The UEA Small RNA Workbench miRProf tool was used without mismatch parameters. The UEA Small RNA Workbench miRCat program was used to identify novel miRNAs [51]. The following parameters were used: genome hits = 16, hit dist = 200, max gaps = 3, max overlap percentage = 80, max percent unpaired = 50, max unique hits = 3, maxsize = 25, min abundance = 1, min energy = -25, min gc = 20, min hairpin length = 60, min paired = 17, min size = 18, orientation percentage = 80, hairpin extension = 100, *p* value = 0.05. If the secondary structure of the sequence met the criteria described by Axtell and Meyers, it was considered a candidate miRNA [52]. The total number of measured values mapped was normalized to the number of reads per million (RPM). The prediction of the new genes was carried out in silico via psRNATarget schema V2 software (https://plantgrn.noble.org/psRNATarget/, accessed on 14 March 2023), with an expectation score of up to 5 and length complementarity of 17 [53].

Quantitative analysis was performed with DESeq2 from the SARTools R package (version 1.49.3) [54]. The dry seed miRNome results from a previous study by Puchta et al. [45] were used as a reference. The raw reads were remapped to the *H. vulgare* reference genome (MorexV3_pseudomolecules_assembly) to utilize the results. On the basis of the new map, the identification of known and novel miRNAs in dry seeds was performed.

To determine the potential role of *H. vulgare* miRNAs in molecular and biological processes, Gene Ontology (GO) annotations of miRNA target genes were extracted from UniProt identifiers [55]. GO categories represented by in silico-predicted miRNA targets were visualized via the g:Profiler toolkit (version 1.2.2) [56]. The results are presented in graphical form via the ggplot2 package (version 3.5.1) for the R package [57].

### 4.5. RT–qPCR Quantification

Reverse transcription–qPCR (RT–qPCR) analyses were performed to validate the results obtained via next-generation sequencing (NGS) [100]. Mature miRNA sequence primers were designed via miRprimer software (version 2.0) [101] and are listed in Supplementary File S1. The miRNAs in each sample with a stable expression level were selected as reference miRNAs via RefFinder. miRNA cDNA synthesis was performed via a Mir-X™ miRNA First-Strand Synthesis Kit (Takara Bio, Kusatsu, Japan) according to the manufacturer’s protocol with 400 ng of miRNA. RT–qPCR was performed via the FastStart Essential DNA Green Master Kit (Roche Diagnostics GmbH, Mannheim, Germany) and a LightCycler 96 thermal cycler (Roche, Mannheim, Germany) according to the manufacturer’s protocols [99]. Three biological and three technical replicates were performed for all analyses.

The expression levels of the target miRNAs were calculated via the ΔΔCt method. Reference miRNAs were selected on the basis of their stable expression in the next-generation sequencing (NGS) data and confirmed via the RefFinder algorithm. This approach has been successfully applied in studies on *Camellia sinensis* and sugarcane buds [102,103].

Two miRNAs, miR159 (M value of 1.19) and miR166 (M value of 1.31), were used as references in this study [34]. Their selection was based on RefFinder analysis results [104]. Each analysis included three biological replicates and three technical replicates per sample.

To detect the expression of miRNA target genes in seeds, the same mRNA samples used for degradome-seq library construction were reverse transcribed via the Maxima H Minus First Strand cDNA Synthesis Kit with dsDNAse (Thermo Fisher Scientific, Waltham, MA, USA).

Real-time PCRs were performed with the following composition: 2 µL of 1× HOT FIREPol EvaGreen qPCR Mix Plus (Solis BioDyne, Tartu, Estonia), 0.25 µM primers, 5.5 µL of water and 2 µL of 20-fold diluted cDNA. The reaction was carried out under the following conditions: initial denaturation at 60 °C for 15 min; 40 cycles of denaturation at 95 °C for 25 s, hybridization of the primers at 60 °C for 25 s, elongation at 72 °C for 25 s and a final elongation at 72 °C for 5 min. The reaction was completed at 4 °C.

The relative expression levels of the genes studied were calculated via 2^−ΔΔCT^ analysis. The reference genes ARF [105] and GAPDH [106] were used for the analysis.

## 5. Conclusions

Previous results by Puchta et al. [34] demonstrated that miRNAs remain intact during long-term dry storage of barley seeds. Both known and novel miRNAs were stable regardless of seed viability or germination capacity or across different germination stages.

In this study, the data provide valuable insights into miRNA dynamics during the germination of seeds with varying viability after long-term storage. Among the identified miRNAs, isomiRs presented sample- and time-specific expression patterns, suggesting that distinct metabolic processes cooccur in seeds with different viability levels during germination.

However, miRNAs from highly conserved plant families—such as miR156, miR159, miR166, miR168, and miR171—showed relatively stable expression over time. This stability suggests their primary role in regulating germination.

Our findings indicate that miRNAs play key roles in processes related to DNA and RNA binding, development, and ribosome regulation during germination. Genes regulated by miRNAs are involved in phosphorylation and phosphorus metabolism, which are essential for the post-translational modification of cell membrane functions and the synthesis of energy carriers necessary for proper germination.

## Data Availability

The data presented in this study are openly available in the NCBI SRA at https://www.ncbi.nlm.nih.gov/bioproject/PRJNA1142182, accessed on 31 July 2024, Accession: PRJNA1142182.

## Supplementary Materials

The following supporting information can be downloaded at https://www.mdpi.com/article/10.3390/ijms26199292/s1.
