# Supplementary material for: Comprehensive Profiling of the miRNome and Degradome Reveals Regulatory Signatures of Seed Aging and Germination
Source: Int J Mol Sci. 2025 Sep 23;26(19):9292. doi: 10.3390/ijms26199292 (PMC12525370; doi:10.3390/ijms26199292)
Supplement: Supplementary file 1 [file ijms-26-09292-s001.zip › Supplementary File S8.pdf]

Supplementary : Details about the *Hordeum vulgare* cultivar Damazy seeds

DESCRIPTION OF THE MATERIAL USED IN THE STUDY

1. OUTLINE OF THE EXPERIMENT

Long term storage seeds *Hordeum vulgare* were imbibed for 24 hours, plant material for testing was collected at 6h, 12h and 24h imbibition. In our previous studies, we focused on changes in the microtranscriptome in dry seeds prior to the imbibition process (doi: 10.3390/ijms22094315) and the results were incorporated into this analysis.

Isolation of genetic material was performed from the embryonic part of dry grains (embryo with scutellum). Material for analysis was obtained from 25 grains per biological replicate. Each grain sample was analyzed in three biological replicates. The embryonic part was excised with a scalpel and then homogenized in liquid nitrogen using a porcelain mortar and pestle. The material thus prepared was used to extract total RNA and miRNA. Figure 1 shows the steps of the experiment.

Isolated miRNA was used to construct sRNA libraries, while degradome-seq libraries were prepared from mRNA enriched on magnetic beads from total RNA.

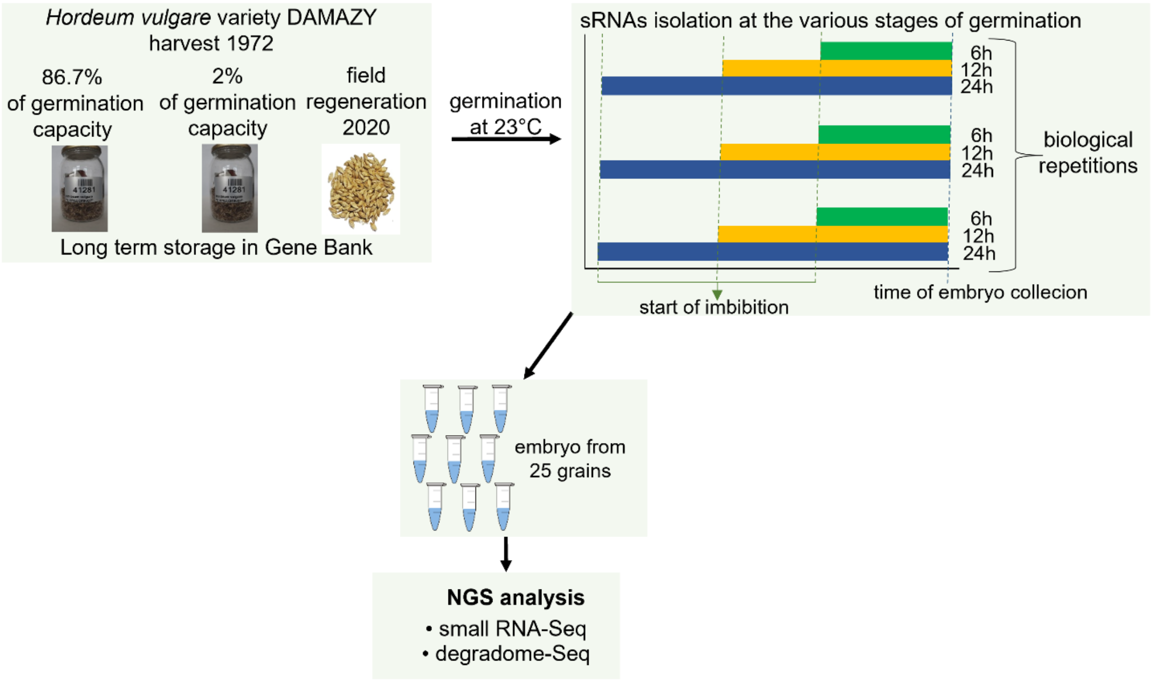

Figure S1. Diagram of the research stages in the experiment conducted

2. PLANT MATERIAL

Grains of *Hordeum vulgare* L. of the Damazy cultivar, which originated in Polanowice and was developed by Mazurkiewicz and Makowski, were used in the study. It was registered in 1969 and removed from the register in 1975. The variety was derived from a cross between varieties of domestic origin. Barley grains from the Plant Breeding Station, Rogaczewo from the 1972 harvest were used for the study. The tested material had a degree of superelite. Superelite is a

grade of cereal that has been recognized in an official evaluation as meeting the requirements for production and quality specified for the basic category, is the third generation preceding seed in the eligible category, and may be intended for the production of seed of the basic category in grade PB/II („https://sip.lex.pl/akty-prawne/dzu-dziennik-ustaw/szczegolowe-wymagania-dotyczace-wytwarzania-i-jakosci-materialu-17330690", [01.09.2021]). Fraction was determined from 1,000 randomly selected grains. For full post-harvest maturity, the grains were stored three months after harvest at 18-25°C. They were then dehydrated in laboratory vacuum dryers (SPT 200) under reduced air pressure (p=iTR) at 40°C from a moisture content of about 15% to a final water content of 2.96%. In order to determine the effect of drying on grain viability, germination tests were conducted. Samples of grains with viability above 95% were placed in airtight, air-filled flasks. The samples were stored for 42 years at ambient temperature.

In 2015, germination capacity tests were conducted again. Seed samples with a high viability of 86.7% and a moisture content of 3.58% and a low viability of 2% and a moisture content of 12.5% were selected for the tests that are the subject of this paper. Seeds with high viability were propagated under field conditions in 2017/2018 and dried to 8%. Reproduced material was used as a control sample in all the experiments described.

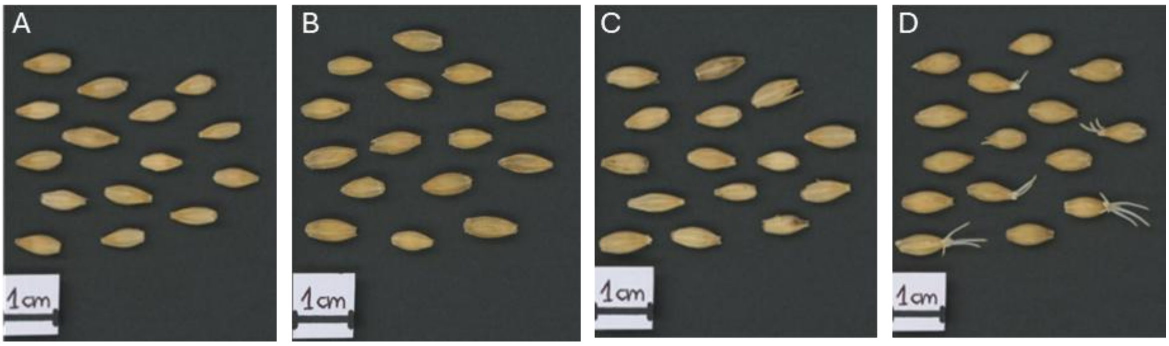

Figure S2. Visual changes occurring in barley grains 0 h (A) and after 6 h (B), 12 h (C) and 24 h (D) of imbibition.

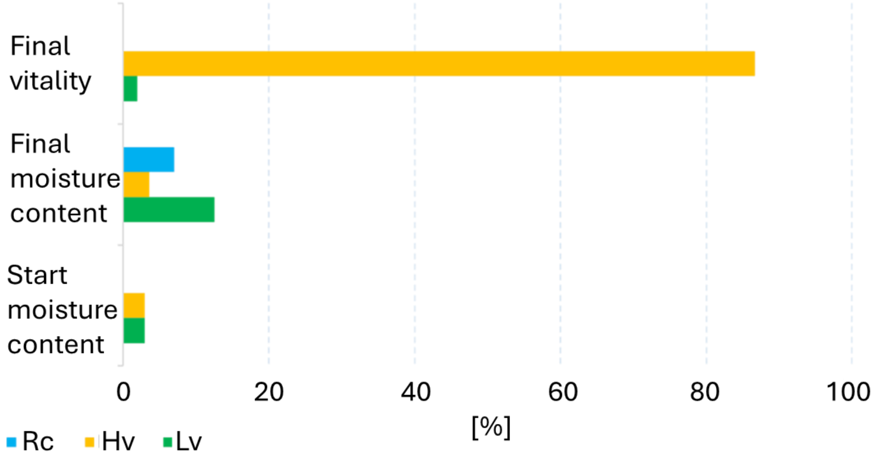

Figure S3. Parameters of the grain used in the study H. vulgare (Hv-high viability seeds; Lv-low viability seeds; Rc- regenerated seeds)

3. GERMINATION TEST

Seeds germination indicators were determined according to the International Rules for Seed Testing (International Seed Testing Association). The germination potential was calculated on days seven, while the germination rate, vigor index, germination index, and dry weight.

Viability tests were performed on tissue paper dishes moistened with water at 20°C for 7 days. Viability was assessed on the basis of % of normal seedlings with developed root, without serious mechanical damage or infection, which show in Figure 4.

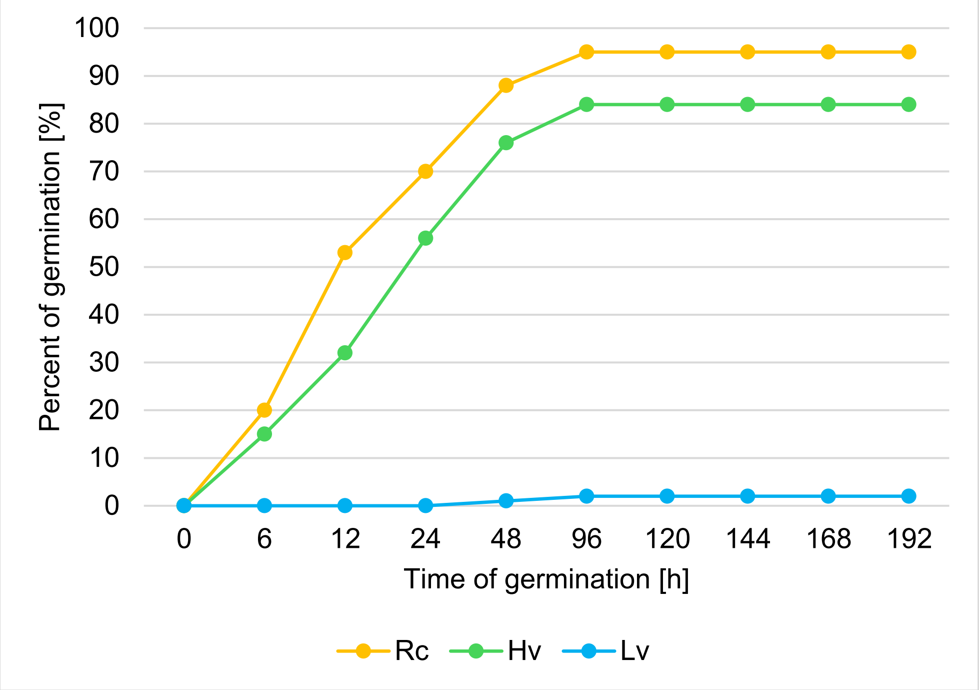

Figure S1. Diagram of percent of germination [%] over time of germination [h] (Hv-high viability seeds; Lv-low viability seeds; Rc- regenerated seeds). The germination test was performed by 192h.

$$Germination\ potential = \frac{n_1}{N} * 100\%$$

n_1:number of seeds germinated during the peak germination period

N: number of tested seeds

$$Germination\ rate\ = \frac{n_2}{N} * 100\%$$

N_2:number of seeds germinated on day 7

N: number of tested seeds

$$Germination\ index = \sum \frac{GT}{DT} (i.e. (\frac{G_1}{N_1} + \frac{G_2}{N_2} ... + \frac{G_n}{N_n})$$

GT: number of seeds germinated on day T

DT: corresponding germination days

G_n: number of seeds germinated on day n

D_n: corresponding germination n days

$$Speed\ of\ emergence = \frac{S_1}{S} * 100\%$$

S_1: Number of germinated seeds at everyday of measurement

S: Number of germinated seeds at final days of measurement

Tabela S1. Seeds parameters measure during germination and viability tests

Rate/Sample	Lv	Hv	Rc
Germination potential	1%	75%	90%
Germination rate	2%	85%	98%
Germination index	0,53	33,3	42,4
Speed of emergence (24*h*)	0,1%	47%	59%

The fresh mass of 24 h barley seeds during germination with seed coat was determined on the laboratory scale (Ohaus Adventurer Pro, USA).

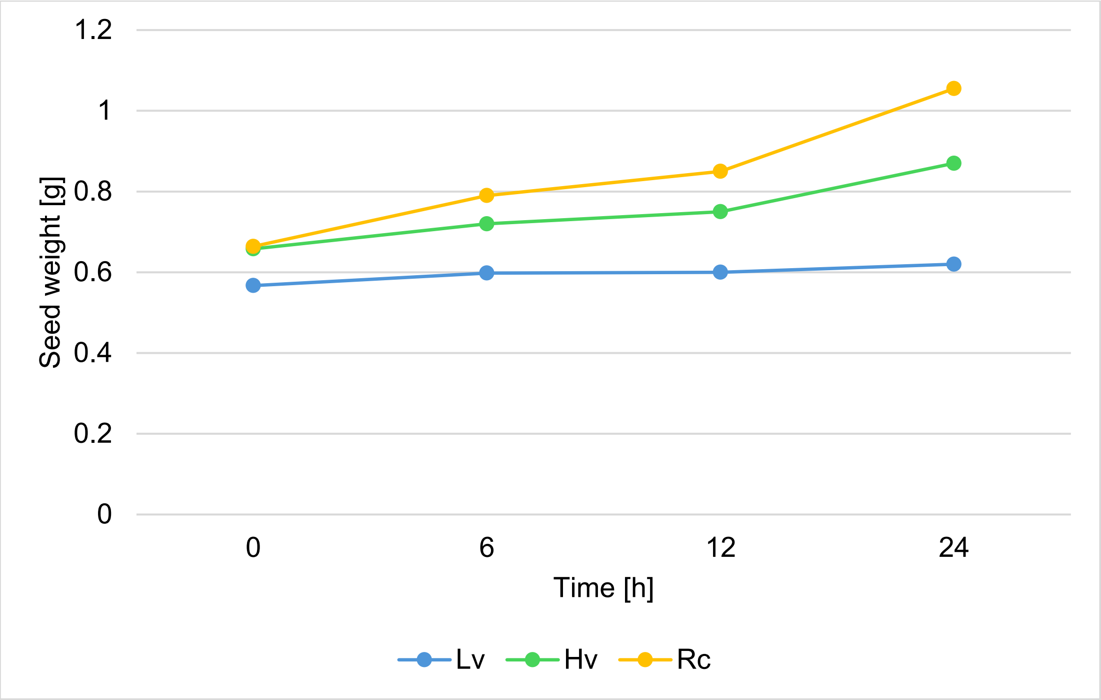

Figure S5. Diagram of seeds dry matter growth [g] over the time [h] (Hv-high viability seeds; Lv-low viability seeds; Rc- regenerated seeds)
