# Supplementary figures and images for: Comprehensive Profiling of the miRNome and Degradome Reveals Regulatory Signatures of Seed Aging and Germination
Source: Int J Mol Sci. 2025 Sep 23;26(19):9292. doi: 10.3390/ijms26199292 (PMC12525370; doi:10.3390/ijms26199292)

## Lv6.1

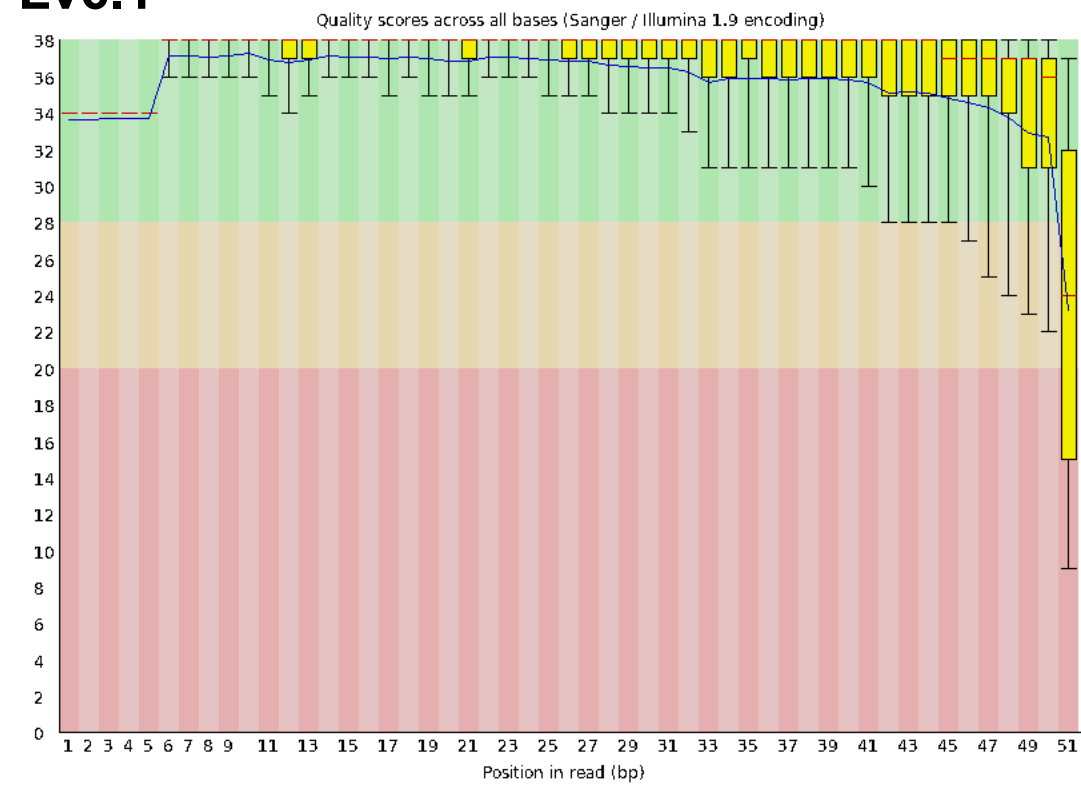

## Lv6.2

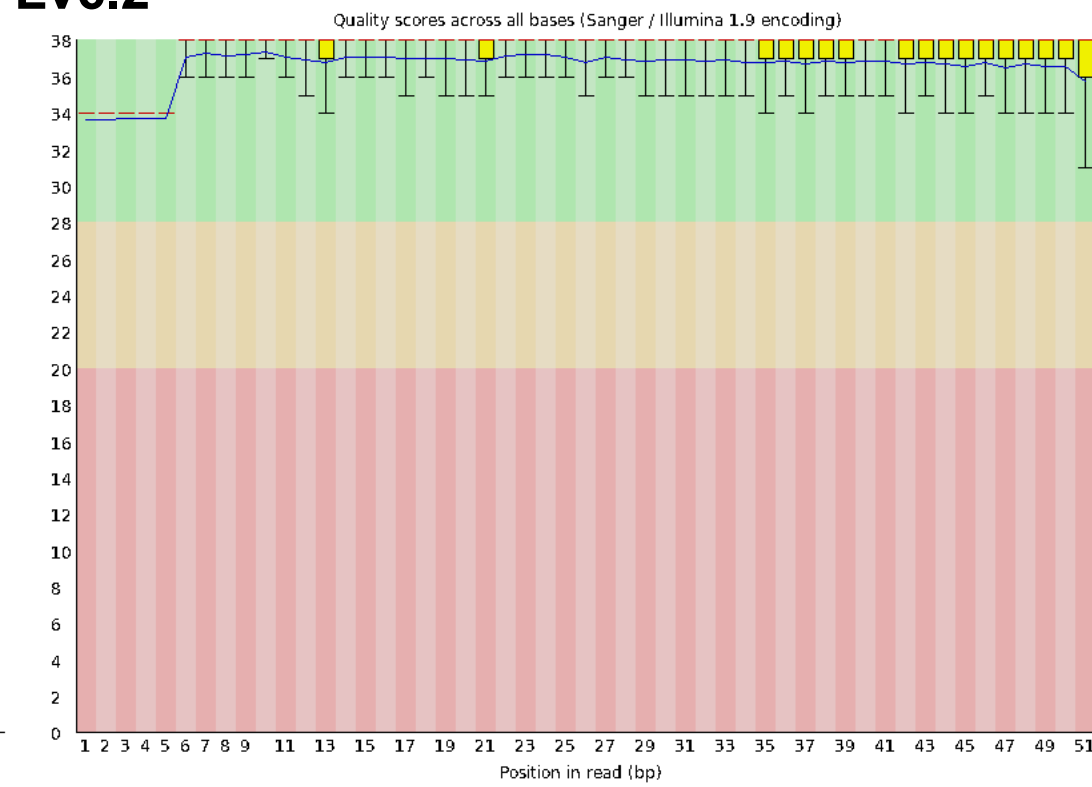

### Lv6.3

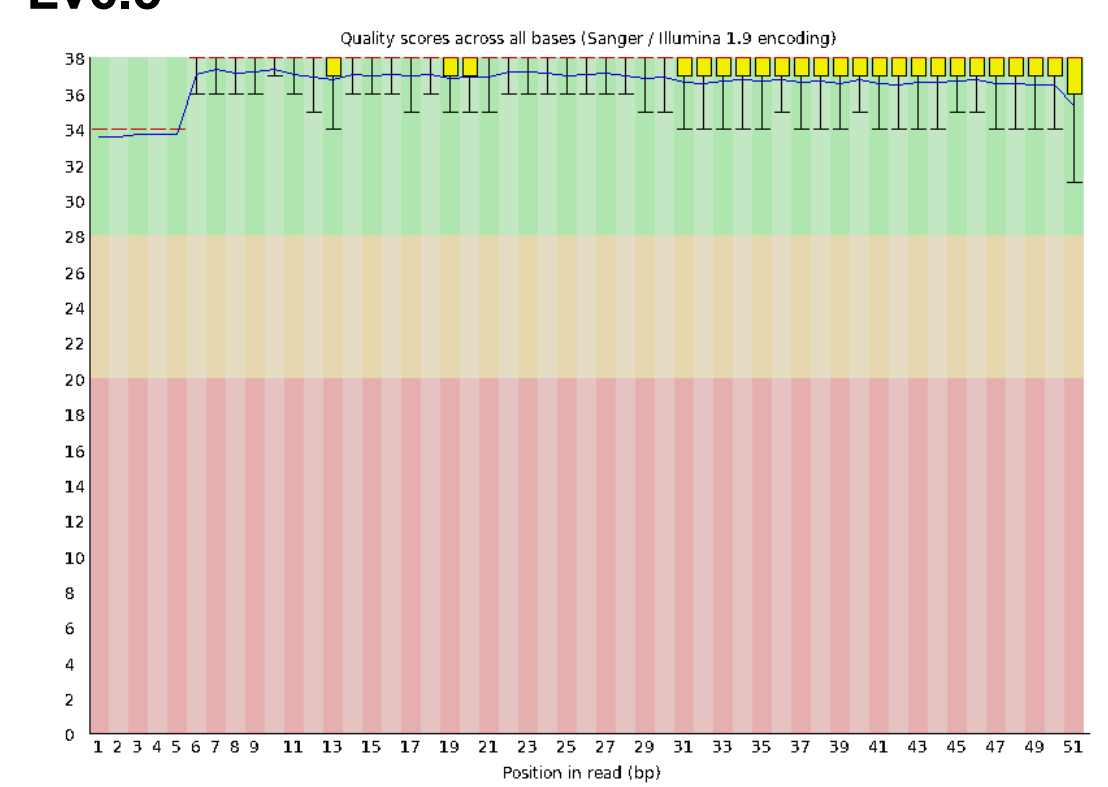

## Lv12.1

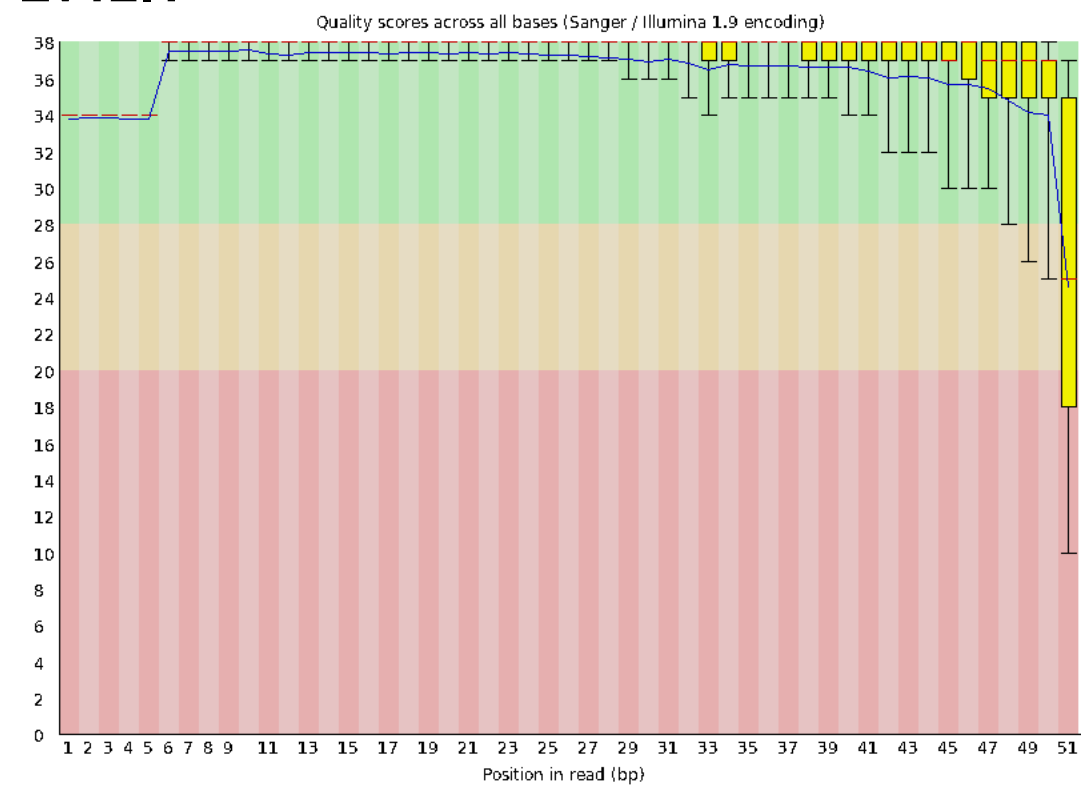

## Lv12.2

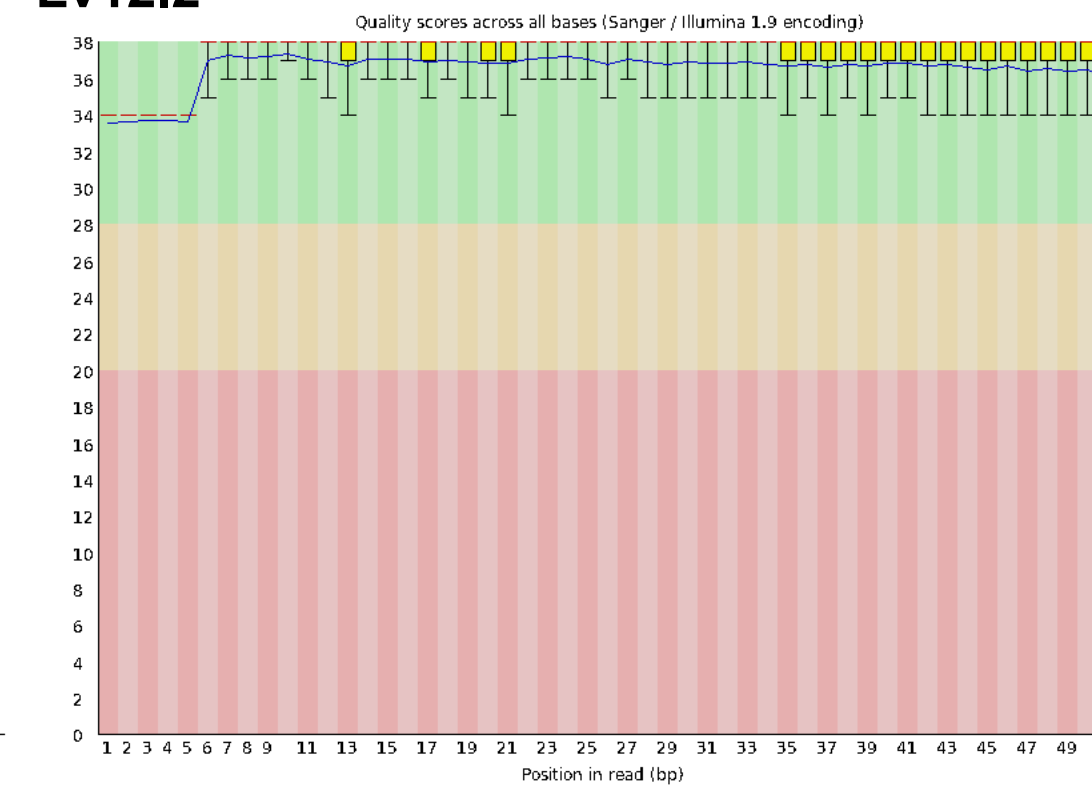

## Lv12.3

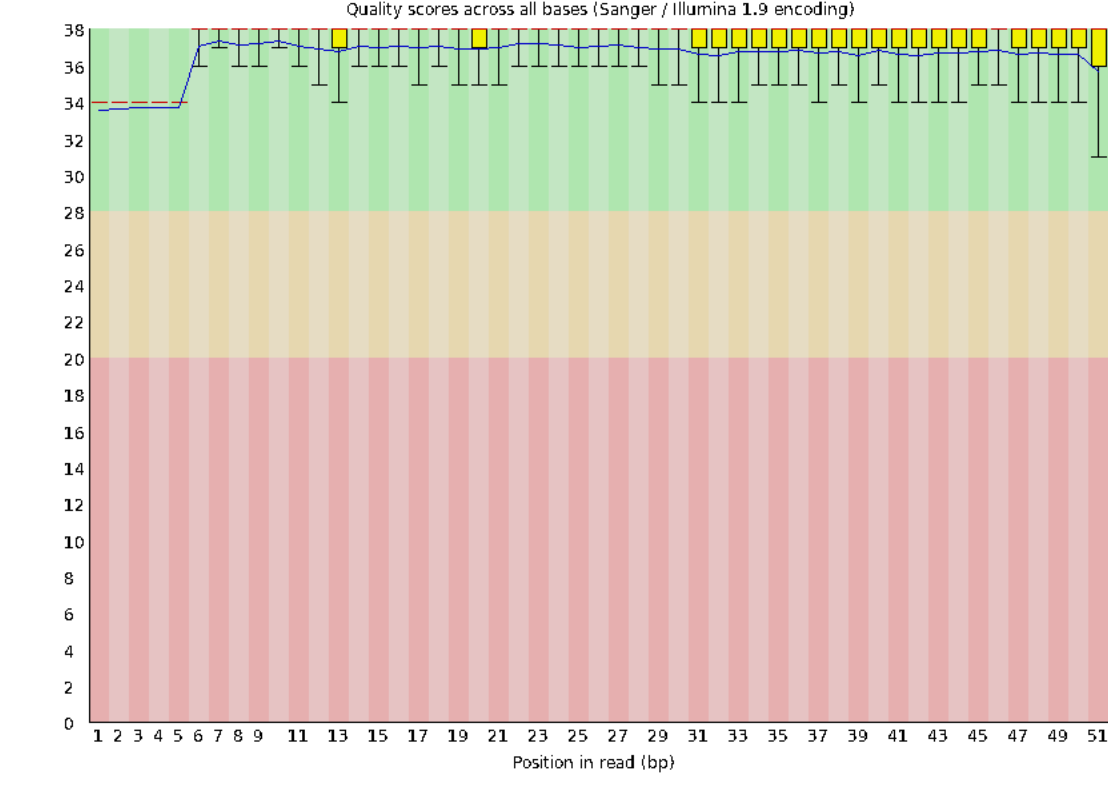

## Lv24.1

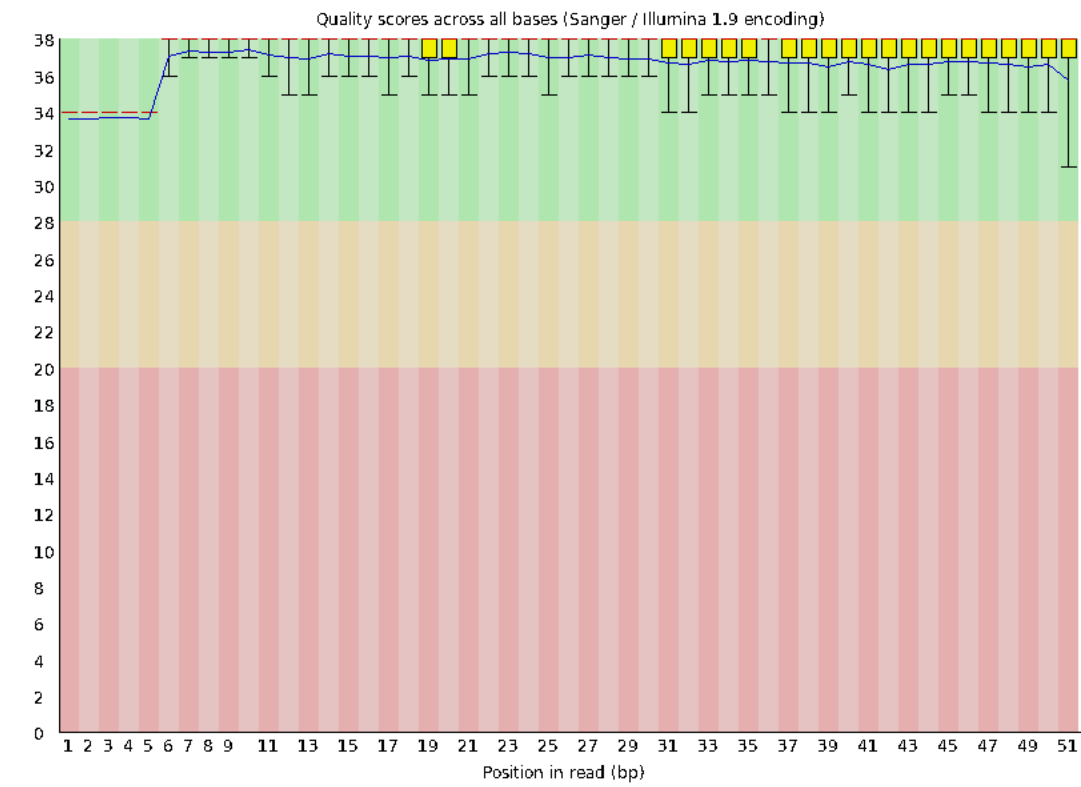

## Lv24.2

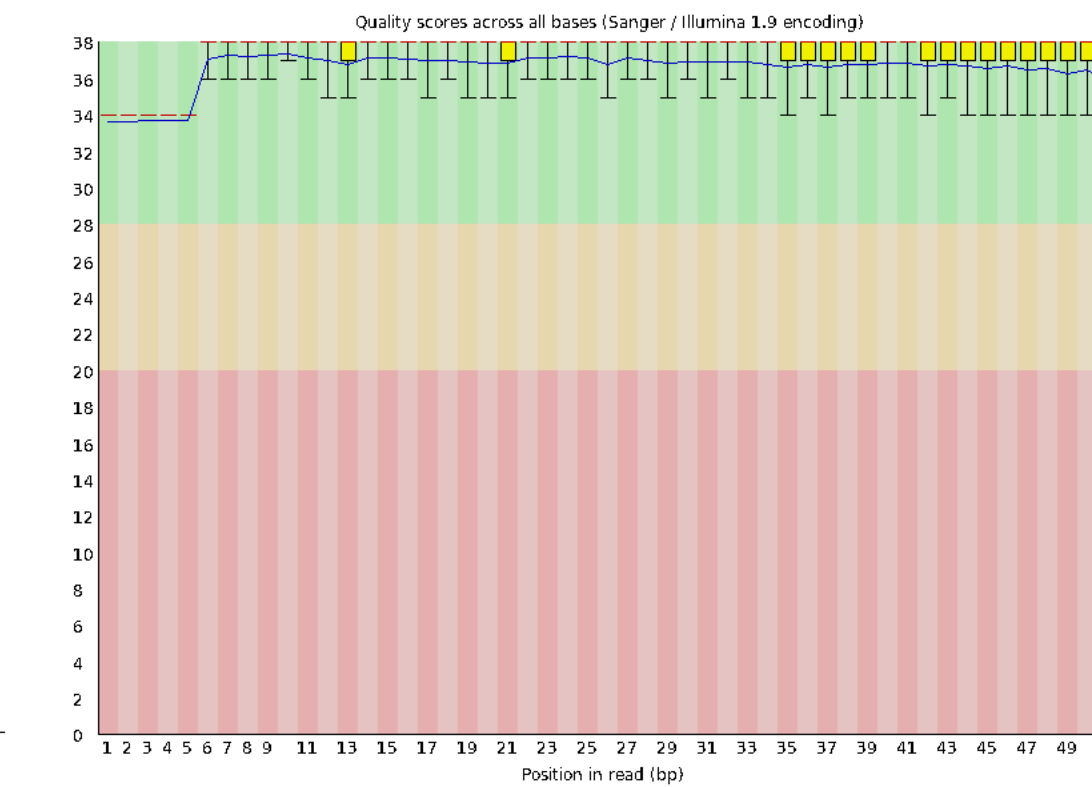

## Lv24.3

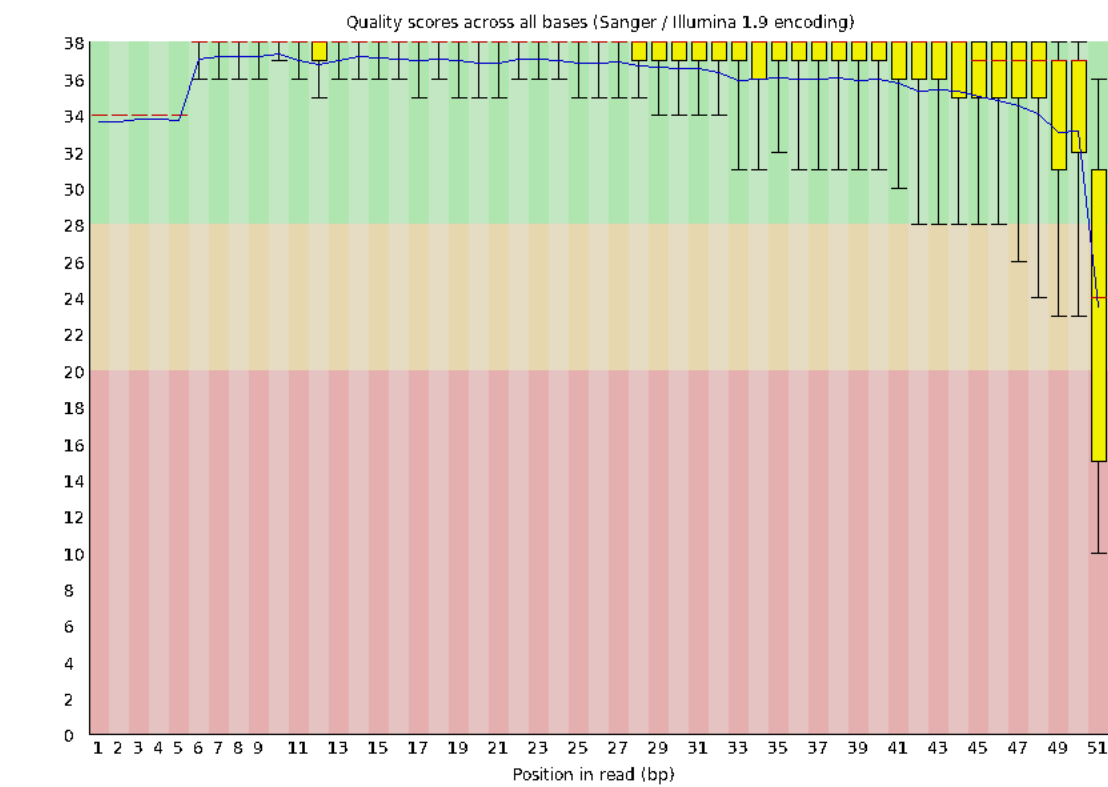

# Hv6.1

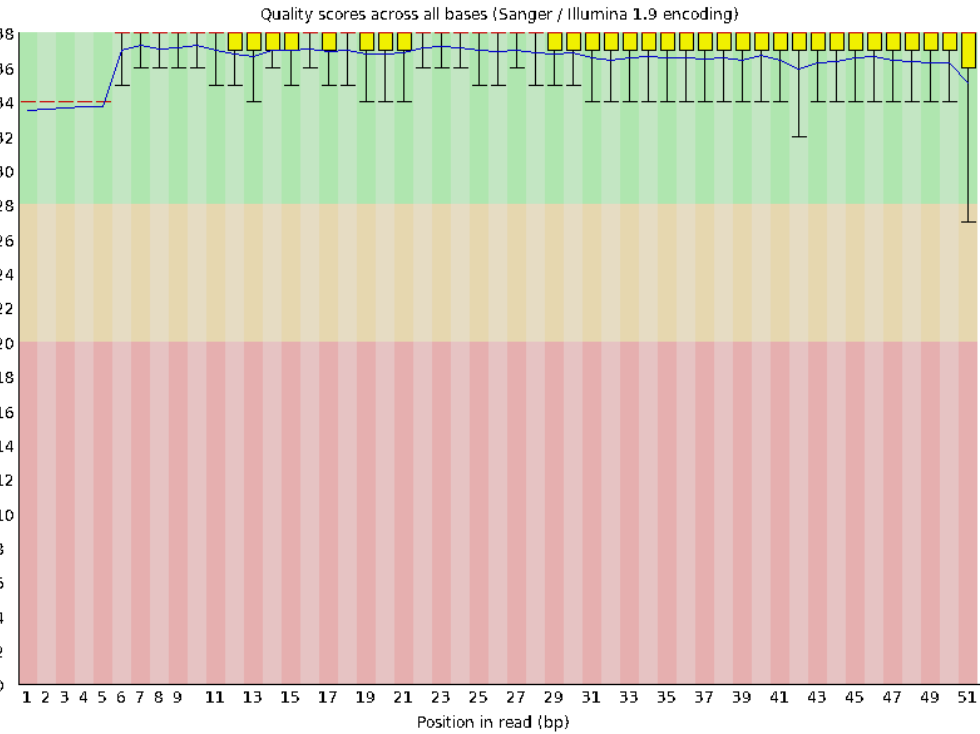

# Hv6.2

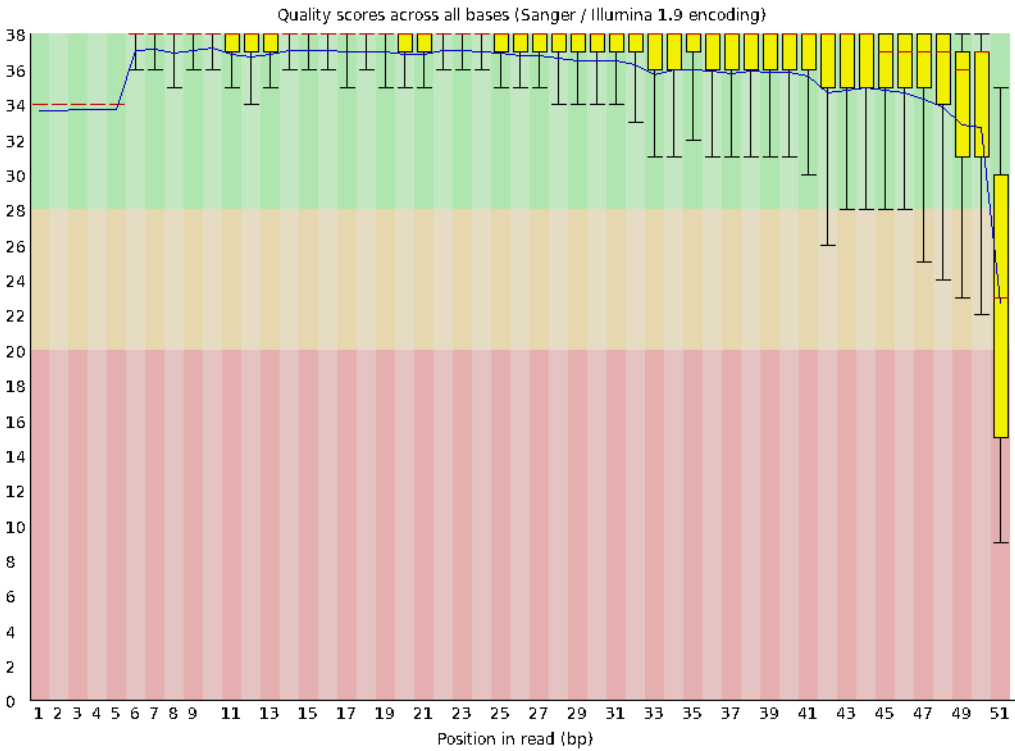

# Hv6.3

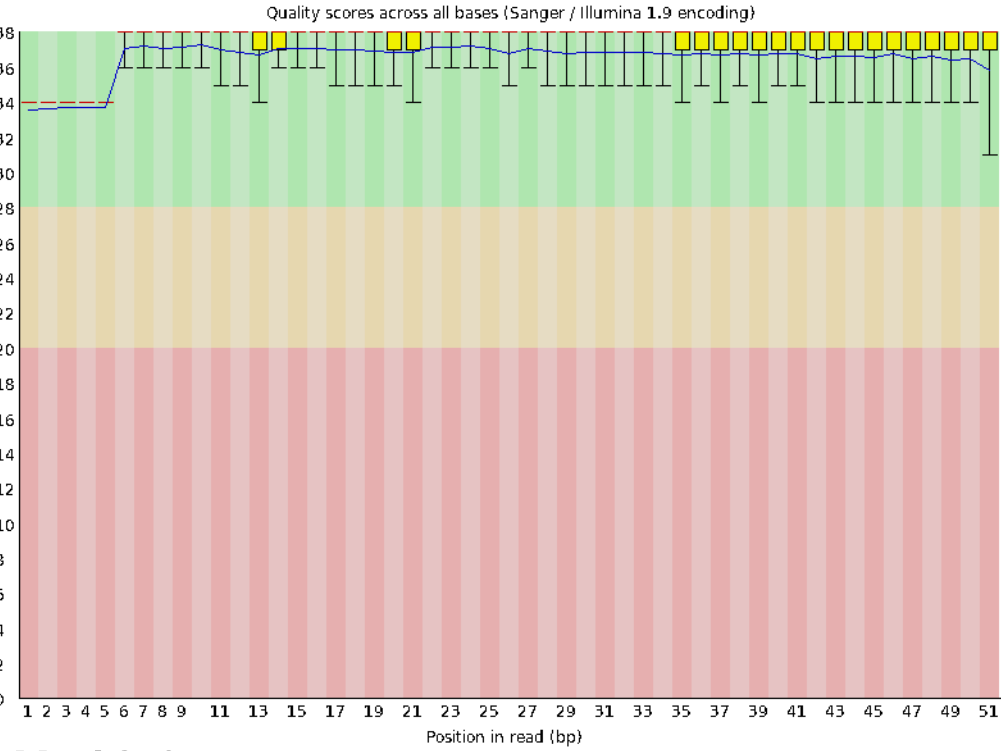

# Hv12.1

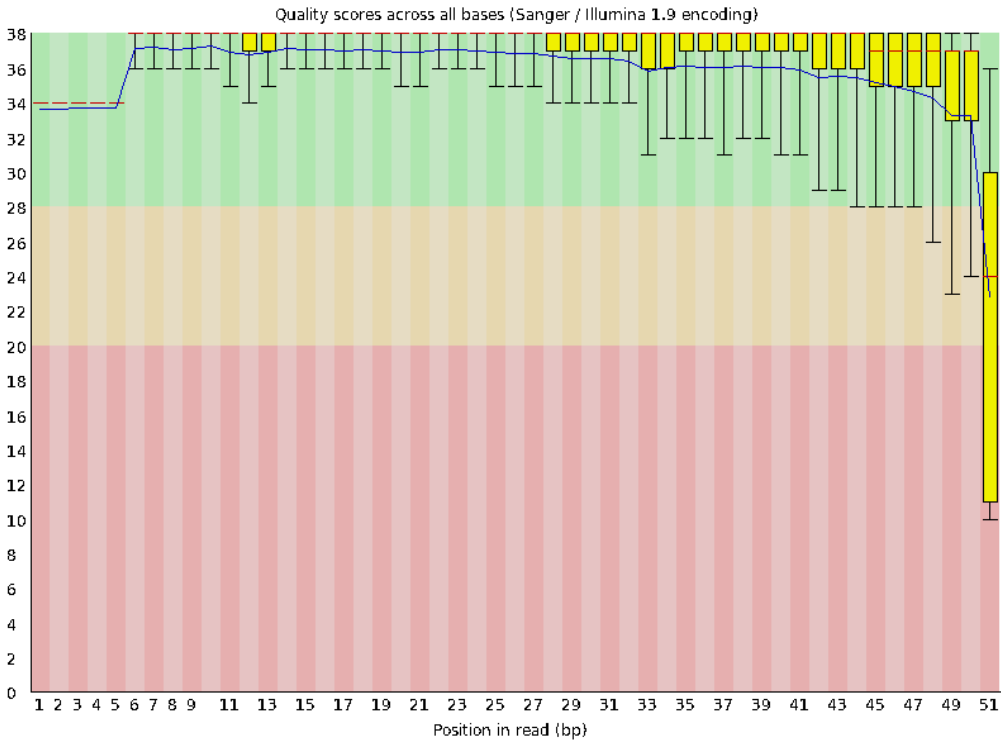

# Hv12.2

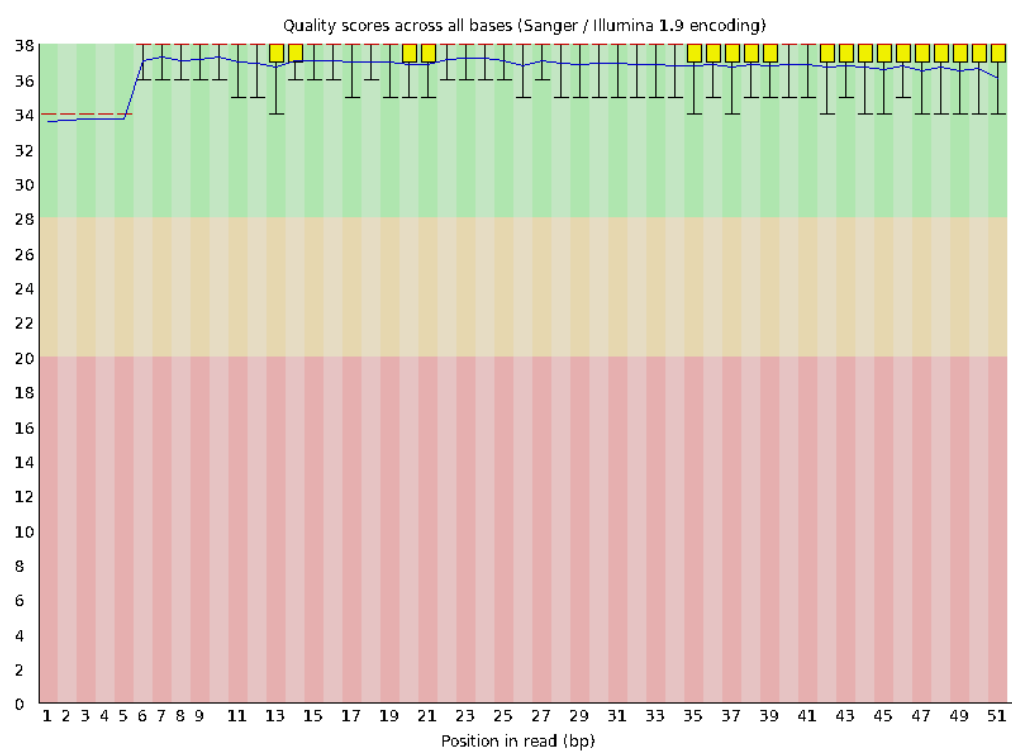

# Hv12.3

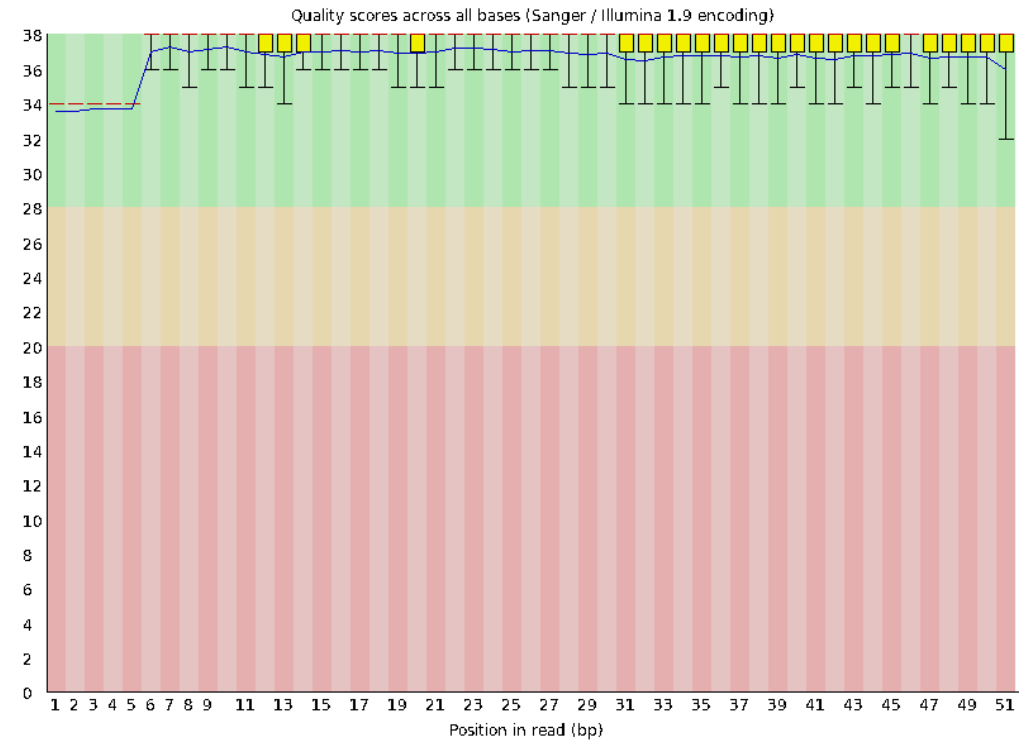

# Hv24.1

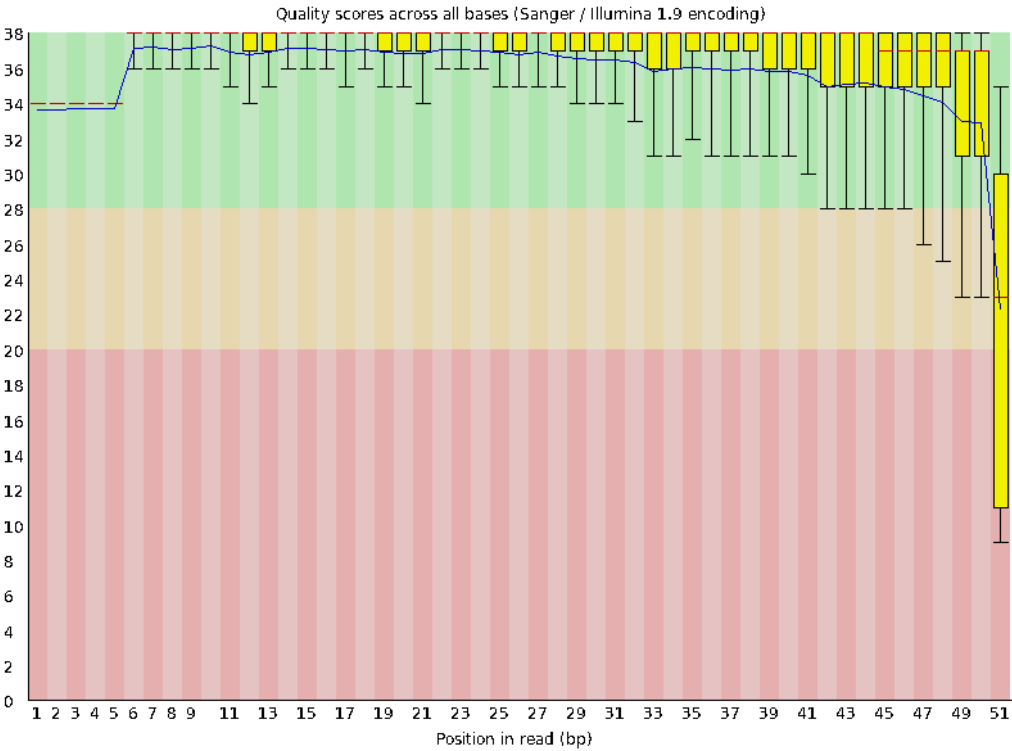

# Hv24.2

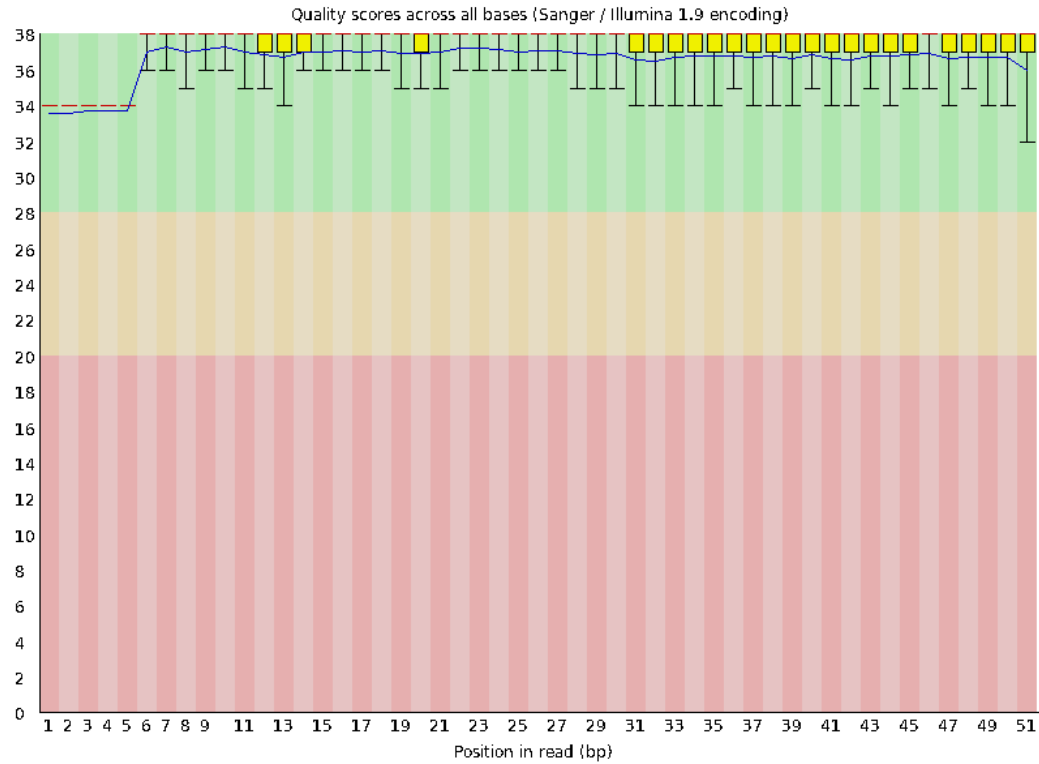

# Hv24.3

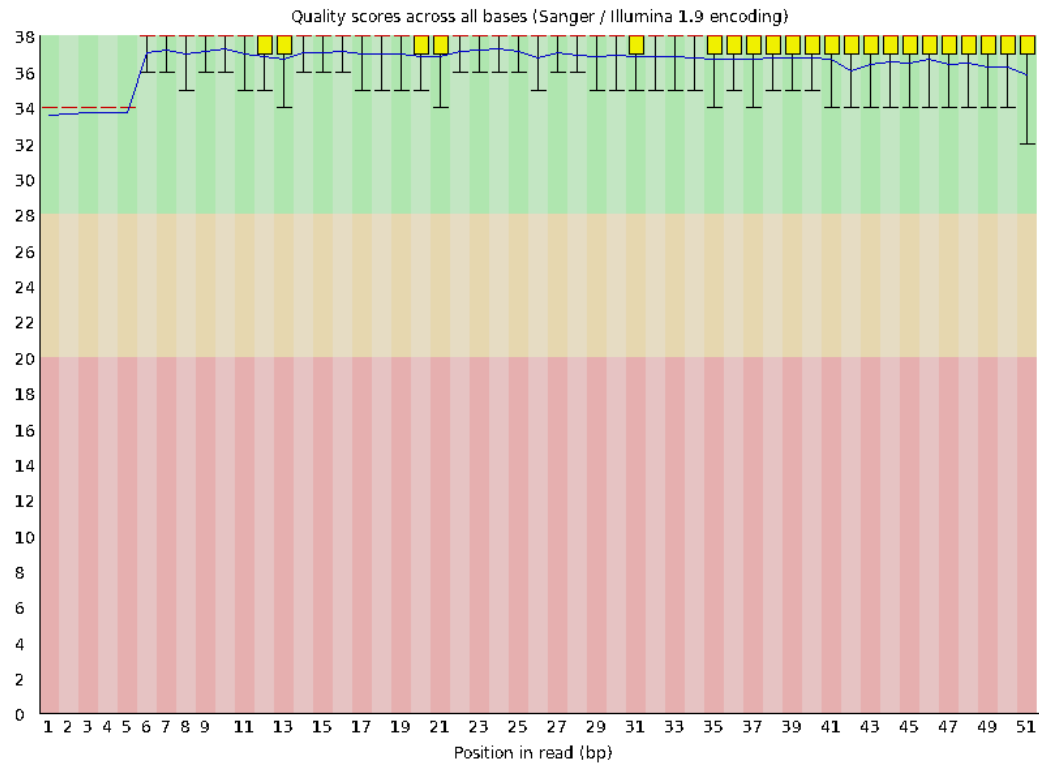

Rc6.1

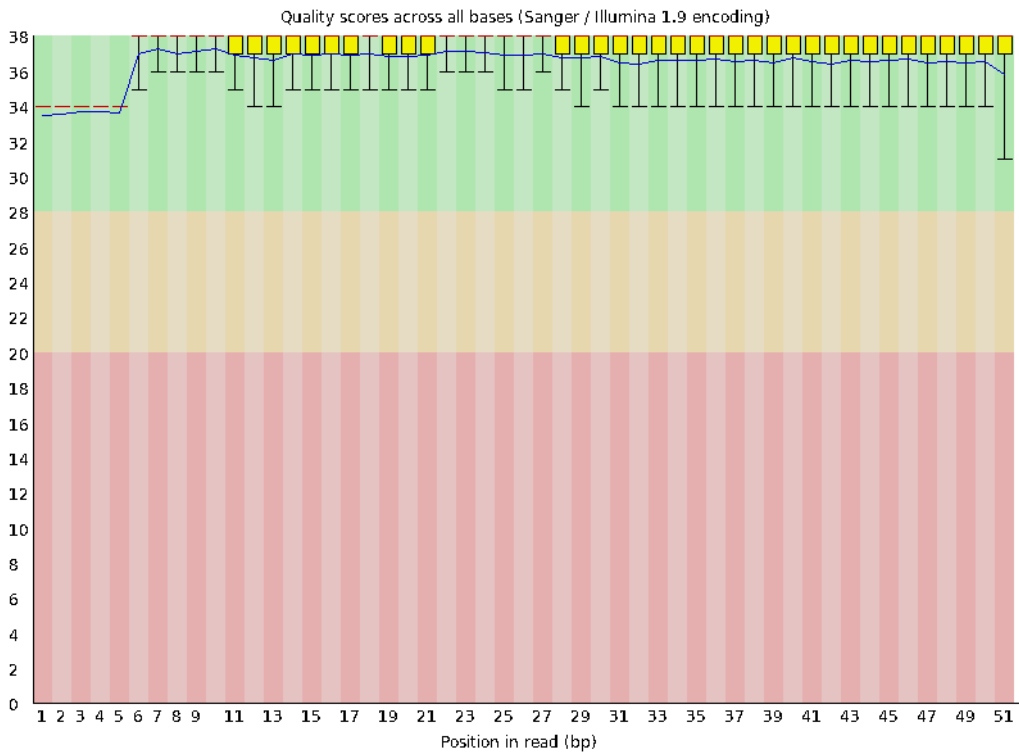

Rc6.2

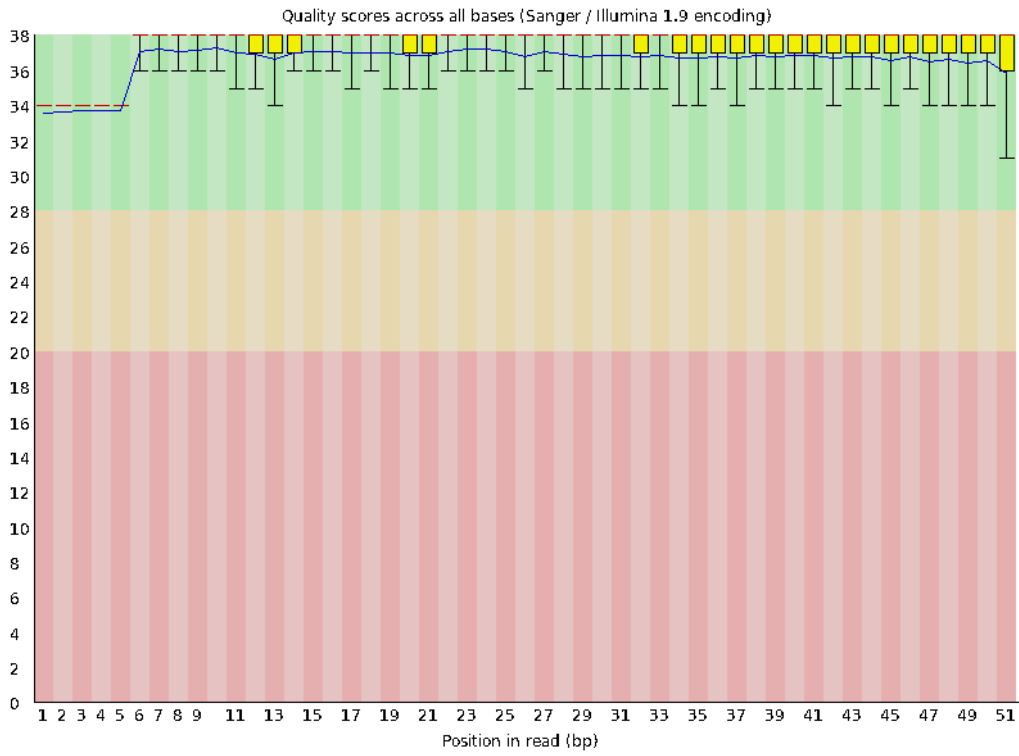

Rc6.3

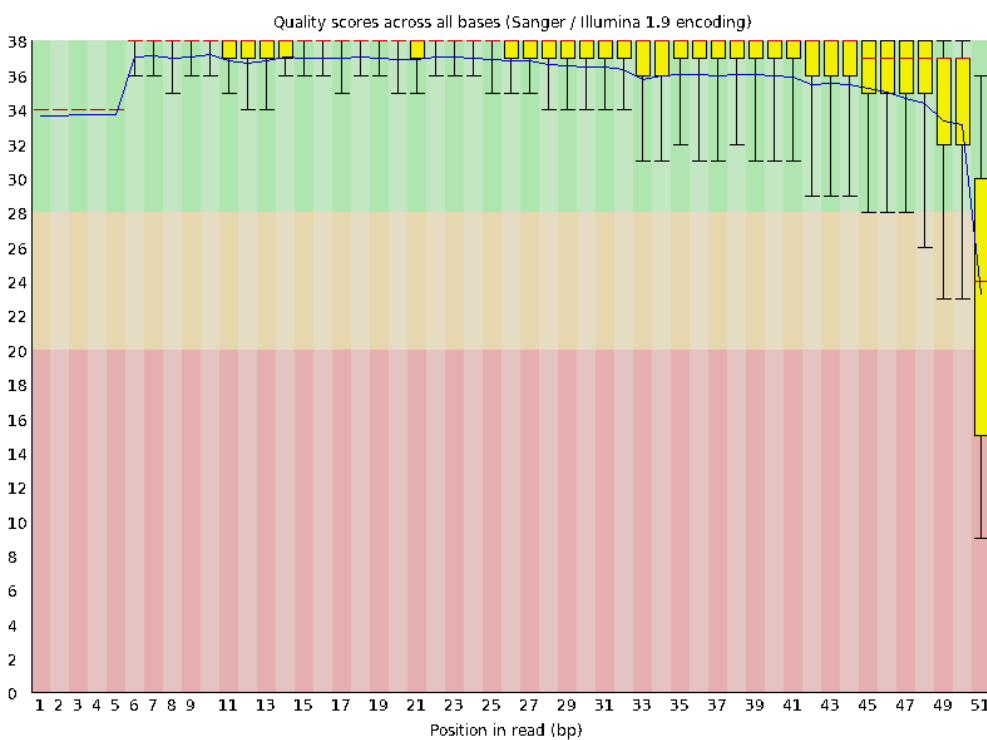

Rc12.1

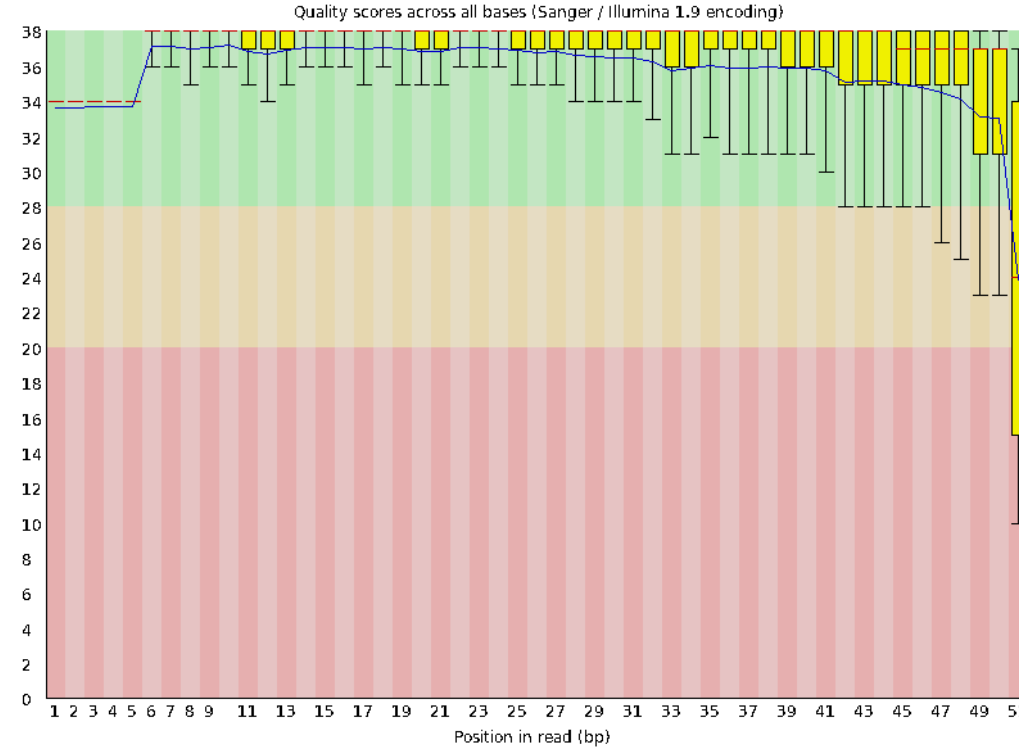

Rc12.2

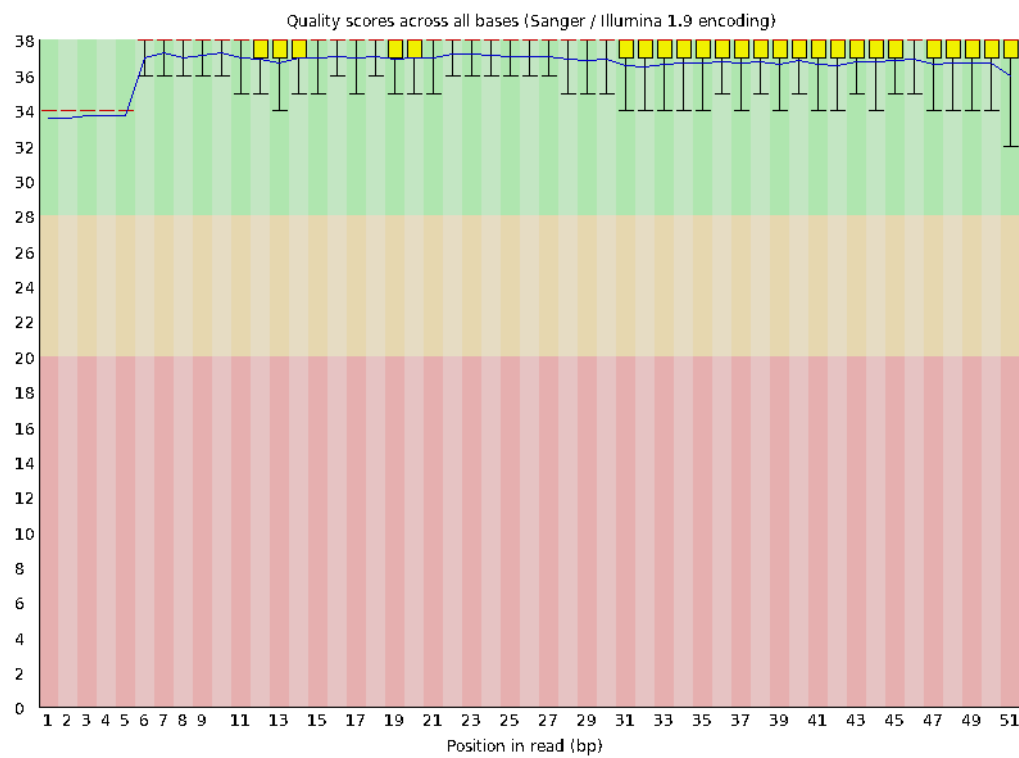

Rc12.3

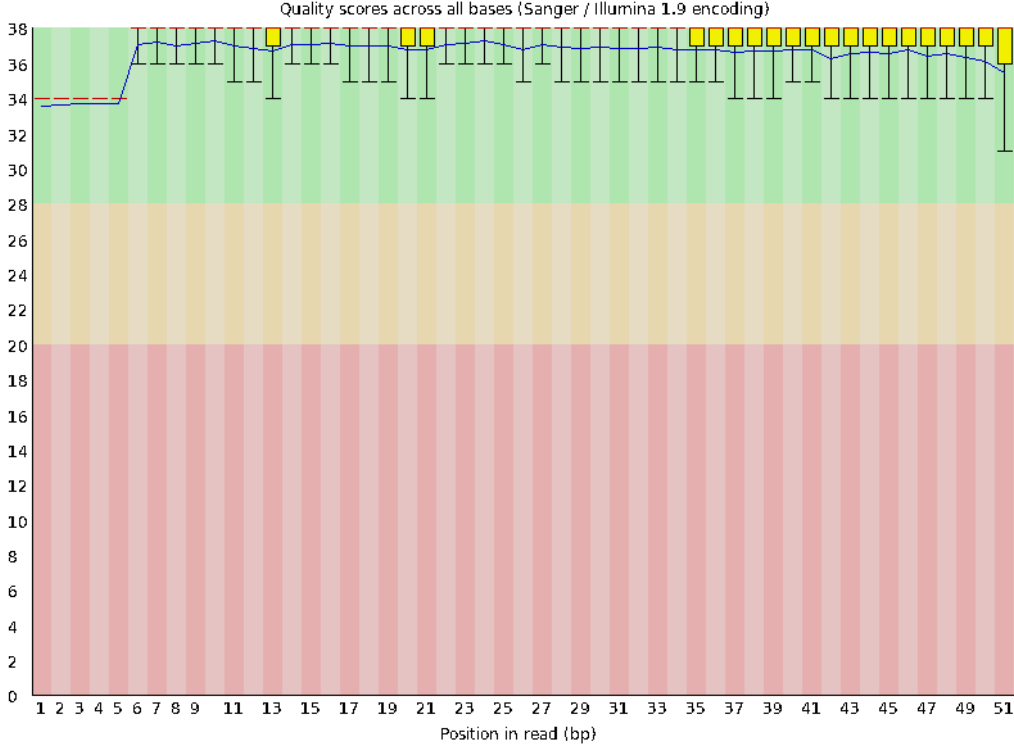

Rc24.1

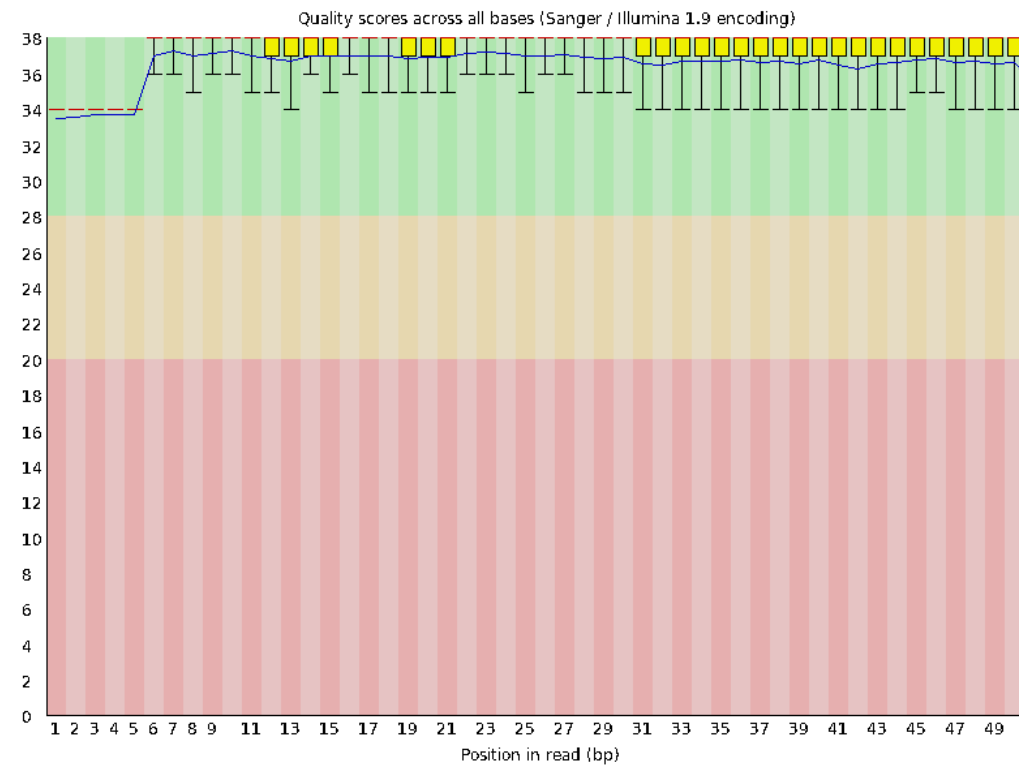

Rc24.2

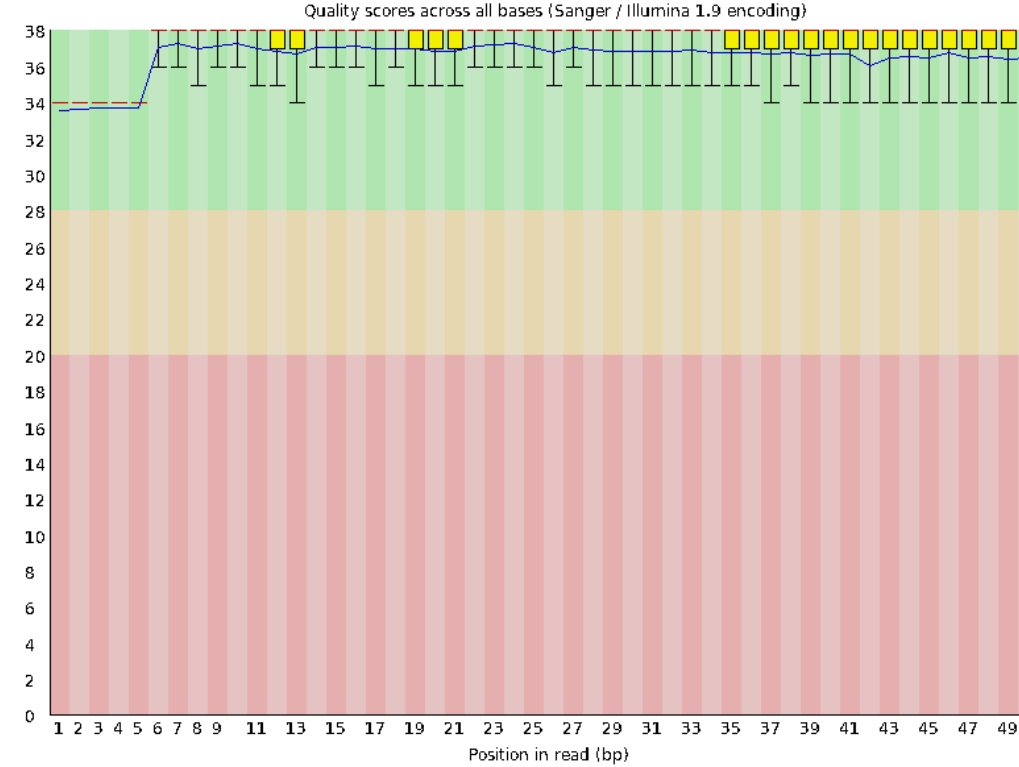

Rc24.3

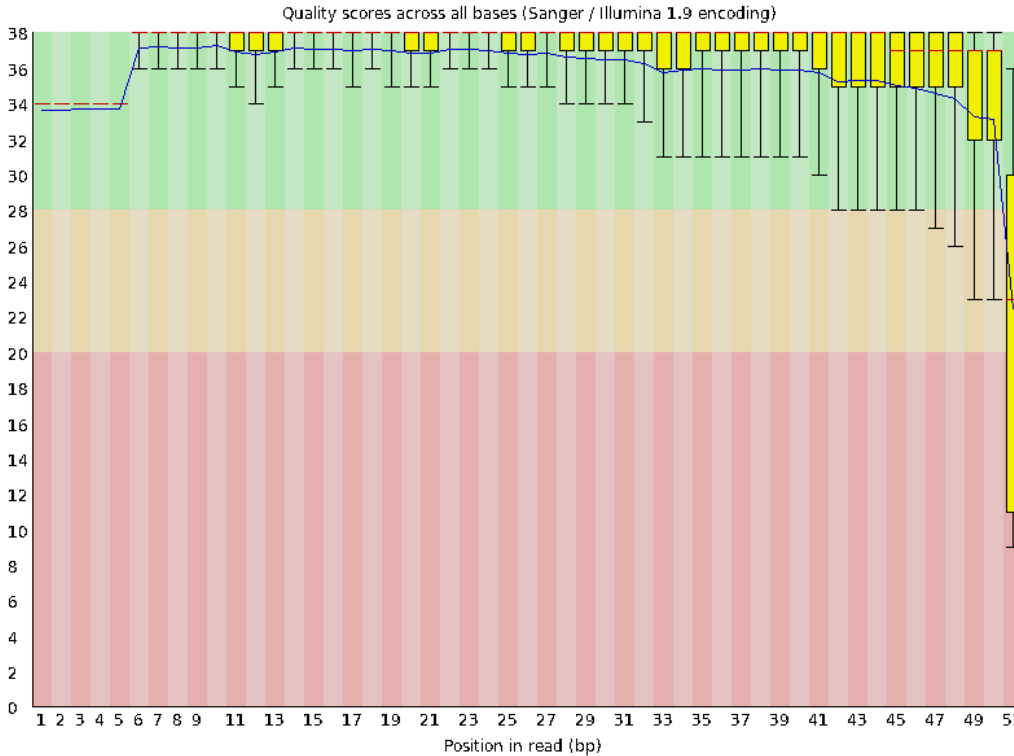

Supplement: Supplementary file 1 [file ijms-26-09292-s001.zip › Supplementary File S9.pdf]
